# Imaging the Landmarks of Vascular Recovery

**DOI:** 10.7150/thno.36022

**Published:** 2020-01-01

**Authors:** Jamila Hedhli, MinWoo Kim, Hailey J. Knox, John A. Cole, Than Huynh, Matthew Schuelke, Iwona T. Dobrucki, Leszek Kalinowski, Jefferson Chan, Albert J. Sinusas, Michael F. Insana, Lawrence W. Dobrucki

**Affiliations:** 1 Department of Bioengineering, University of Illinois at Urbana-Champaign, Urbana, IL; 2 Department of Electrical and Computer Engineering, University of Illinois at Urbana-Champaign, Urbana, IL; 3 Department of Chemistry, University of Illinois at Urbana-Champaign, Urbana, IL; 4 SimBioSys, Inc., Champaign, IL; 5 Beckman Institute for Advanced Science and Technology, Urbana, IL; 6 Department of Internal Medicine, Yale University School of Medicine, New Haven, CT; 7 Department of Medical Laboratory Diagnostics, Medical University of Gdansk, Poland; 8 Biobanking and Biomolecular Resources Research Infrastructure Poland (BBMRI.PL), Gdansk, Poland

**Keywords:** Angiogenesis, hypoxia, hindlimb ischemia, ^99m^Tc-NC100692, ^ 99m^Tc-BRU-5921, perfusion, functional recovery, Power Doppler imaging.

## Abstract

**Background**: Peripheral arterial disease (PAD) is a major worldwide health concern. Since the late 1990s therapeutic angiogenesis has been investigated as an alternative to traditional PAD treatments. Although positive preclinical results abound in the literature, the outcomes of human clinical trials have been discouraging. Among the challenges the field has faced has been a lack of standardization of the timings and measures used to validate new treatment approaches.

**Methods**: In order to study the spatiotemporal dynamics of both perfusion and neovascularization in mice subjected to surgically-induced hindlimb ischemia (n= 30), we employed three label-free imaging modalities (a novel high-sensitivity ultrasonic Power Doppler methodology, laser speckle contrast, and photoacoustic imaging), as well as a tandem of radio-labeled molecular probes, ^99m^Tc-NC100692 and ^99m^Tc-BRU-5921 respectively, designed to detect two key modulators of angiogenic activity, *α_V_β_3_* and HIF-1α , via scintigraphic imaging.

**Results**: The multimodal imaging strategy reveals a set of “landmarks”—key physiological and molecular events in the healing process—that can serve as a standardized framework for describing the impact of emerging PAD treatments. These landmarks span the entire process of neovascularization, beginning with the rapid decreases in perfusion and oxygenation associated with ligation surgery, extending through pro-angiogenic changes in gene expression driven by the master regulator HIF-1α , and ultimately leading to complete functional revascularization of the affected tissues.

**Conclusions**: This study represents an important step in the development of multimodal non-invasive imaging strategies for vascular research; the combined results offer more insight than can be gleaned through any of the individual imaging methods alone. Researchers adopting similar imaging strategies and will be better able to describe changes in the onset, duration, and strength of each of the landmarks of vascular recovery, yielding greater biological insight, and enabling more comprehensive cross-study comparisons. Perhaps most important, this study paves the road for more efficient translation of PAD research; emerging experimental treatments can be more effectively assessed and refined at the preclinical stage, ultimately leading to better next-generation therapies.

## Introduction

Peripheral arterial disease (PAD), the progressive narrowing of the non-cerebral and non-coronary arteries, is estimated to affect around 27 million people in Europe and North America 1. Its prevalence has become so great that despite inherent difficulties in diagnosing it—around half of cases are asymptomatic—newly-identified PAD patients account for approximately 35% of hospitalizations in the United States 2. Moreover, because PAD is so frequently missed until its later stages, it is also associated with a high risk of debilitating complications, including claudication pain, tissue necrosis, and ulceration often leading to foot amputation 3. While the public health challenges posed by PAD continue to mount, clinical approaches to its management have been slow to evolve. In chronic cases, treatment generally focuses on lifestyle factors, including cessation of tobacco use, changes in diet, and increased physical exercise. In critical cases, surgical approaches can be employed (such as vascular bypass or angioplasty), but with relatively limited long-term efficacy 4. Despite all of this, PAD research has been extremely active for decades. Between 1981 and 2007, an estimated 155,000 scientific articles were published on the subject 5, begging the question of why so few discoveries are being translated from bench to bedside.

Many proposed PAD therapies have revolved around methods to enhance angiogenic neovascularization 6. Angiogenesis is a dynamic and complex multistep process, requiring endothelial proliferation and differentiation, an interplay of various pro- and anti-angiogenic factors, and interactions among endothelial cells, the extracellular matrix (ECM), and smooth muscle cells 7. During reductions in physiological oxygen levels, the master transcriptional activator, hypoxia inducible factor1-α (HIF-1α), induces expression of a number of angiogenic mediators including vascular endothelial growth factor (VEGF), fibroblast growth factor (FGF), granulocyte- and granulocyte macrophage colony stimulating factors (G- and GM-CSF), and the VEGF receptors, Flt-1 (VEGFR-1) and Flk-1 (VEGFR-2) 8-11, each of which has been considered as a therapeutic target for the management of PAD 12. As newly established blood vessels begin to grow, endothelial cells upregulate the expression of integrins, including *α_V_β_3_*, *α_V_β_5_*, and *α_5_β_1_*, as they migrate and modulate tube formation 13. Not only are these integrins critical for cell adhesion and motility, they also serve as important signal transducers, interacting with a diverse cast of both intra- and extracellular signaling molecules that tightly regulate their behavior.

The complexity of the molecular and cellular processes that drive angiogenesis make its modulation extremely challenging. Redundancies, nonlinearities, and feedback loops in the angiogenic signaling pathways—features that impart an inherent robustness to the process—also make it difficult to predict *a priori* whether an intervention affecting one step will actually produce significant enhancements in later steps. In an outstanding recent review of the challenges facing translational therapeutic angiogenesis, Iyer and Annex raise the example of an adenovirus engineered to express HIF-1α that, in clinical trials, failed to result in enhanced expression of its downstream regulatory targets 12,14. This should serve as a cautionary tale; it is not enough to show that a therapy impacts its intended target (*e.g.* HIF-1α expression); it must also be shown that the therapy's impact extends down the angiogenic signaling cascade, effecting targets like VEGF or *α_V_β_3_*.

The most commonly used preclinical PAD models—namely mice that have undergone unilateral femoral artery ligation—pose several challenges of their own. First, it is difficult to model years or decades of cardiovascular risk-factors—tobacco use, poor diet, obesity, *etc.*—in weeks- or months-old mice. And while PAD develops slowly in humans, with progressive tissue injury beginning long before symptoms even present, in preclinical models its effects are induced suddenly via surgery. Second, as noted by Waters *et al.*, most mouse lines tend to recover from femoral artery ligation in a matter of weeks even without treatment, meaning that there exists a limited window of time during which the effectiveness of a treatment can be meaningfully ascertained 15. Measure too early, and the treatment might not yet show efficacy; measure too late, and the difference between treated and untreated animals might be too small to discern. Finally, vascular recovery proceeds at different rates and through different mechanisms in different regions of the body. Measuring vascular function at only a single location (*e.g.* via ankle brachial pulse index) can lead to a myopic view of the recovery process as a whole.

Although progress is yet to be made in faithfully reproducing the effects of decades of hard-living on the arteries of a mouse, significant strides are being made in understanding the spatial, temporal, and biomolecular complexities of the body's response to vascular occlusion. What has been needed are methods that enable a holistic assessment of multiple biomarkers—both molecular and physiological—over the entire duration of vascular recovery. To that end, the past several years have seen an explosion of new methods for the non-invasive *in vivo* imaging of peripheral vasculature. These include major advances aimed at imaging blood perfusion using ultrasound (US) 16,17, photoacoustic imaging (PA) 18-21, and laser speckle contrast imaging (LSCI) 22, as well as quantitative molecular imaging approaches using radiolabeled probes targeted at specific angiogenic biomarkers such as matrix metalloproteinases, VEGF isoforms, and *α_V_β_3_* integrin 23-31. While each of these imaging modalities are powerful on their own, and capable of providing functionally relevant windows into molecular and physiological processes in both animals and humans, their greatest potential lies in their combined use as part of a broader multimodal imaging strategy.

The purpose of this article is to set forth one of the first multidimensional platforms designed to track the progression of several different measures associated with vascular occlusion and neovascularization. The platform employs power Doppler ultrasound imaging (US), photoacoustic imaging (PA), laser speckle contrast imaging (LSCI), and targeted molecular scintigraphic imaging. Using these modalities, we serially image tissue perfusion and oxygenation, hypoxia inducible factor 1α (HIF-1α) activity, and *α_V_β_3_* integrin activity in the ischemic tissues of mice that have undergone femoral artery ligation. Combined, these markers paint a highly detailed time-resolved portrait of vascular recovery, with several clearly-defined “landmarks” along the way. These landmarks begin with the earliest molecular response to hypoxia and follow the course of both the deep and shallow tissues through their reperfusion and reoxygenation. Perhaps most importantly, these landmarks can form the basis of a standardized vocabulary for describing neovascularization. Researchers adopting similar serial multimodal imaging strategies will be able to describe the impact of promising new PAD interventions in terms of the onset, strength, and duration of a series of interrelated biological events that span the entire recovery process. Not only does this benefit individual researchers developing the next generation of PAD therapies, it aids the entire community by enabling more comprehensive comparisons across studies and between interventions with the potential to improve reproducibility of PAD research outcomes.

### Translational Perspective

Despite decades of intense research and numerous clinical trials, the options available for the treatment and management of PAD remain largely unchanged. One of the challenges the field has faced in translating discoveries from the bench to the clinic has been a lack of standardization in the biomarkers and endpoints used to evaluate the efficacy of new therapeutic interventions in the pre-clinical phase. This has rendered cross-study comparisons of new treatments extremely challenging and limited the entire field's ability to detect and refocus its efforts on the most-promising classes of candidates. In this article we present a new multimodal imaging platform designed to track changes in a number of key measures of vascular function and recovery in a mouse model of PAD. Using this platform, we define a series of landmarks that span from the first few minutes after the onset of vascular occlusion all the way to complete functional recovery several weeks later. These landmarks can serve as a new framework for understanding and comparing the therapeutic efficacies of future PAD treatments, leading to more efficient translation with fewer resources wasted on ineffective treatments.

## Materials and Methods

**Figure 1:**
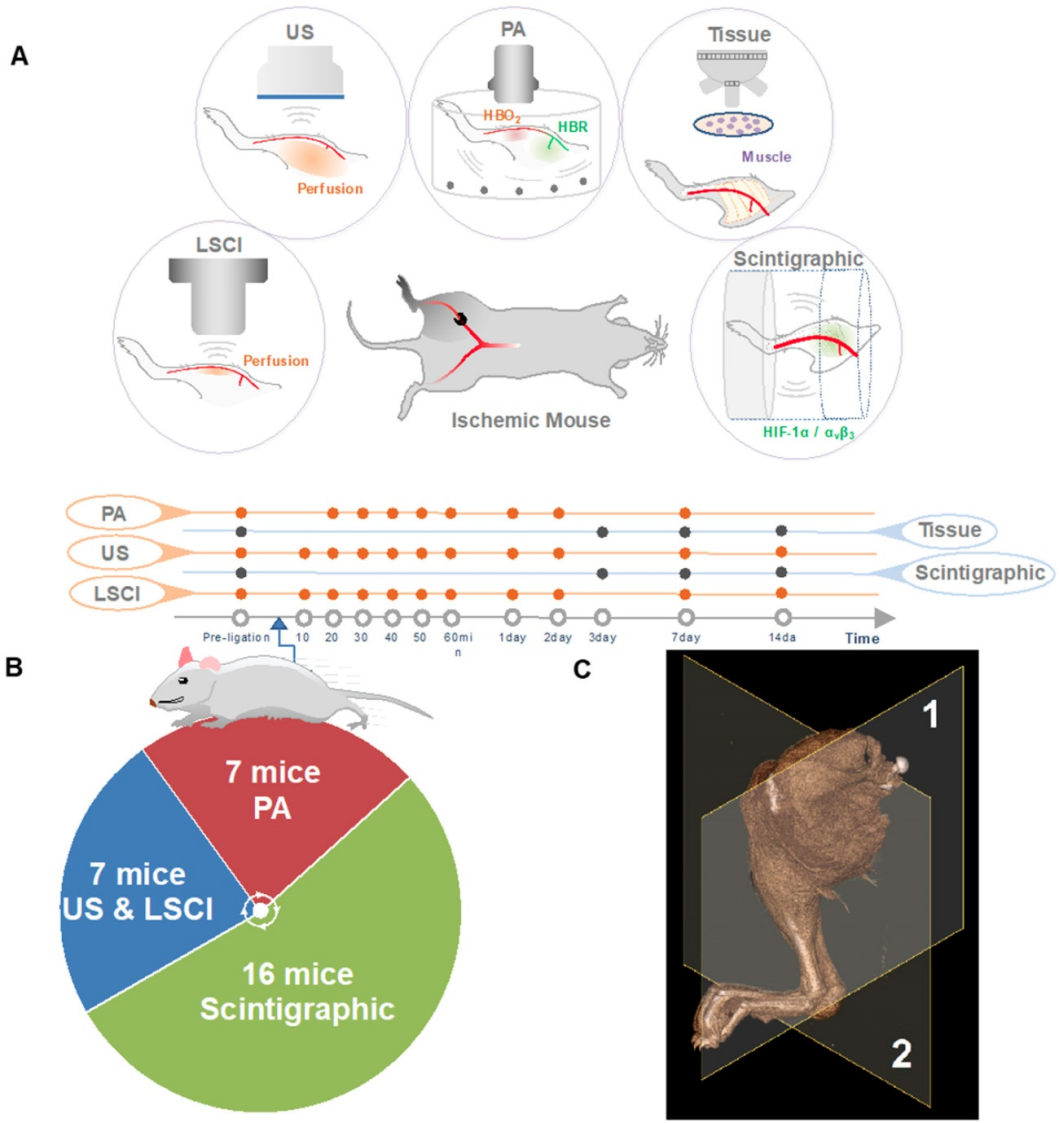
Study overview. (A) The right femoral artery was ligated to mimic PAD. The animals were serially evaluated for angiogenesis (targeted at *α_V_β_3_*) and hypoxia (targeted at HIF-1α), measures of blood perfusion (using US, LSCI, and PA). At days three, seven and 14, skeletal muscle tissue was extracted for immunohistochemistry experiments (denoted Tissue). (B) The animal numbers used in various experiments. (C) Imaging plane for ultrasonic Power-Doppler imaging (deep-tissue perfusion), photoacoustic imaging (PA, blood oxygenation) represented by (1), Imaging plane for scintigraphy imaging (angiogenesis, hypoxia), laser speckle-contrast imaging (LSCI, shallow-tissue perfusion) represented by (2).

The research plan is outlined in Figure 1. Briefly, 4 to 6-week-old mice were subjected to right femoral artery ligation in order to induce peripheral ischemia. Animals were imaged serially with several modalities at various time points to investigate spatiotemporal changes within the ischemic microenvironment.

### Animals

All experiments were performed according to the guiding principles of the American Physiological Society and approved by the Institutional Animal Care and Use Committee.

Induction of unilateral hindlimb ischemia via surgical ligation of the right femoral artery was performed on 30 male black C57BL/6 mice (Charles River Laboratories, US) according to procedures described previously 32. Additional details are presented in Supplementary Methods section *Animals*.

### Ultrasound Imaging

A subset of animals (n=7) were used for perfusion assessment employing a novel ultrasonic power Doppler method recently described in 16. The mice were placed in a supine position on a heating pad set to 37 °C with both hindlimbs extended. They were then imaged prior to ligation surgery and at a series of post-operative time points (10, 20, 30, 40, 50, and 60 minutes, and 1, 2, 7, and 14 days). Additional details on the acquisition and analysis of our US data are presented in Supplementary Methods sections *Ultrasound Imaging* and *Perfusion Trend Analysis*.

### Laser speckle contrast imaging

The same seven (n=7) animals assessed using ultrasonic Power-Doppler imaging were also scanned using laser speckle contrast imaging. LSCI was performed prior to ligation surgery, and at the same series of post-operative time points used for US imaging (10, 20, 30, 40, 50, and 60 minutes, and 1, 2, 7, and 14 days). Additional details are presented in Supplementary Methods sections *LSCI Imaging* and *Perfusion Trend Analysis*.

### Photoacoustic imaging

Another subset of seven (n=7) mice was used for PA imaging. Each animal was imaged at 20, 30, 40, 50, and 60 minutes, as well as 1, 2, and 7 days after surgery. Additional details on the acquisition and analysis of our PA data are presented in Supplementary Methods sections *Photoacoustic Imaging* and *Blood Oxygenation Analysis*.

### Serial Scintigraphic Imaging

To assess the *in vivo* specificity of the ^99m^Tc-labeled tracers, as well as their suitability for PAD imaging, a subset of animals (n=16) were subjected to serial scintigraphic imaging. Images were acquired before, and at 3, 7, and 14 days after ligation surgery. Details on the procedures used are described in Supplementary Methods section *Scintigraphic Imaging*. The quantification of ROIs is presented in (see Figure 5 B).

### Histological Validation

A subset (n=12) of the animals that underwent scintigraphic imaging were then used for histological characterization. After each imaging session (pre-surgery, 3, 7, and 14 days post-surgery), three animals were euthanized, and their gastrocnemius/soleus muscle complexes were excised for subsequent analysis of skeletal muscle vascularity, HIF-1α expression, and probe (NC100692) specificity. Details can be found in Supplementary Methods section *Histological Validation.*

## Results

### A Multimodal Platform for Imaging Vascular Recovery

The primary goal of monitoring the circulatory system using multiple imaging techniques is to find an accurate, temporally-resolved, and non-invasive strategy that can help describe vascular occlusion and neovascularization holistically, using a range of molecular and physiological biomarkers. Our platform includes methods focused on imaging perfusion (US, LSCI), blood oxygenation (PA), and the activity of the key angiogenic markers HIF-1α and *α_V_β_3_* (scintographic imaging).

**Figure 2:**
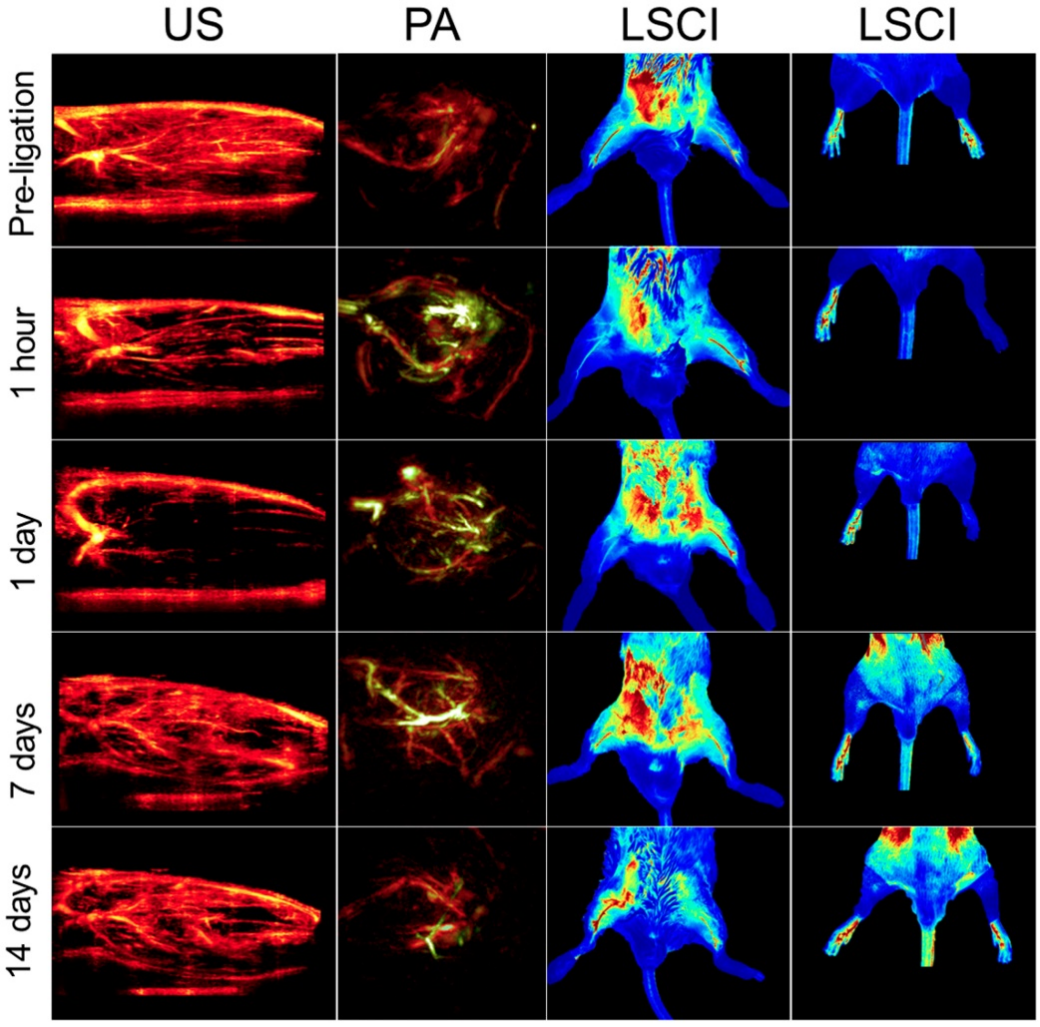
Representative multimodal imaging data presented at a series of time points (prior to and following ligation). Each column shows how a different circulatory parameter changes over time as the mice recover. The first column highlights perfusion as a series of 10 mm × 15 mm vertical cross-sections of ischemic tissue imaged using US. Prior to ligation, perfusion generally appears strong, except for the distal regions of the limb where the signal appears to be diminished primarily due to acoustic attenuation. The second columns show PA images of the ischemic tissues excited at 750 nm (for HbR detection, green color) and 850 nm (for HbO2 detection, red color). The third column represented LSCI images of the legs in a horizontal view. The right hindlimbs (appearing on the left side of the images) underwent femoral artery ligation, while the left hindlimbs were used as controls within each animal. The fourth column represents LSCI images of feet.

Individually, each method can output only one measurand, and each modality suffers from its own inherent limitations. Together, however, their strengths and shortcomings are complementary. Scintigraphic imaging, for example, which relies on radiolabeled probes to image specific biomolecules, requires a relatively long time between subsequent imaging sessions in order to allow for the radioactivity to decay. US, LSCI, and PA, on the other hand, can be performed repeatedly or even continuously, but they do not allow for the same type of molecular specificity. Similarly, US and LSCI, both of which can image perfusion, have very different penetration depths, resolutions, and imaging contrasts (see Table 1), and can thus be used to image different regions of the ischemic tissue (see Figure 2).

**Figure 3:**
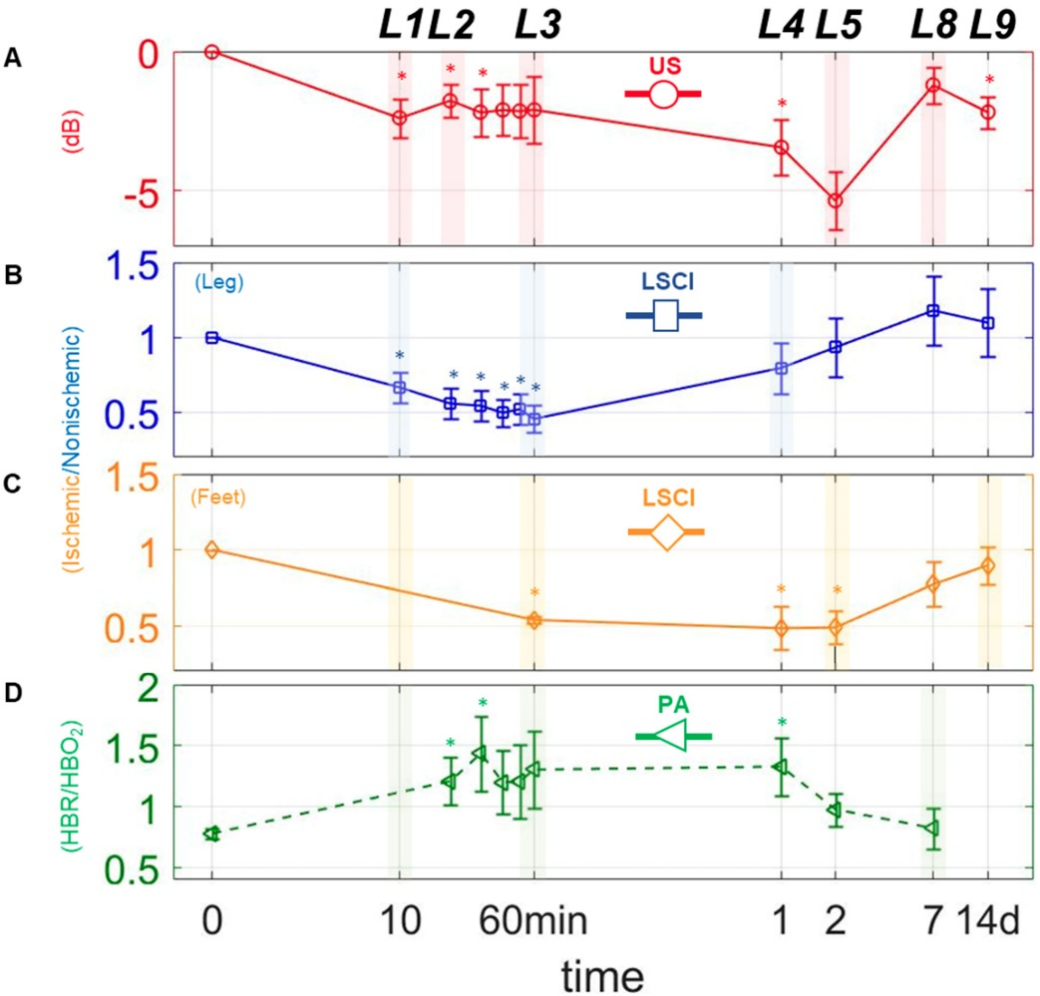
Changes in perfusion and hypoxia over a logarithmic timeline. Points marked (◦) and (□) are for perfusion estimates using US and LSCI, respectively. Points marked (□) indicate the ratio of deoxygenated to oxygenated hemoglobin (HbR/HbO2) as measured via PA. (A)-(D), represents plots of each measure individually. The mark (∗) above each point indicates a statistically significant (p < 0.05) difference from the preligation state. L above each time point indicates a landmark.

**Figure 4:**
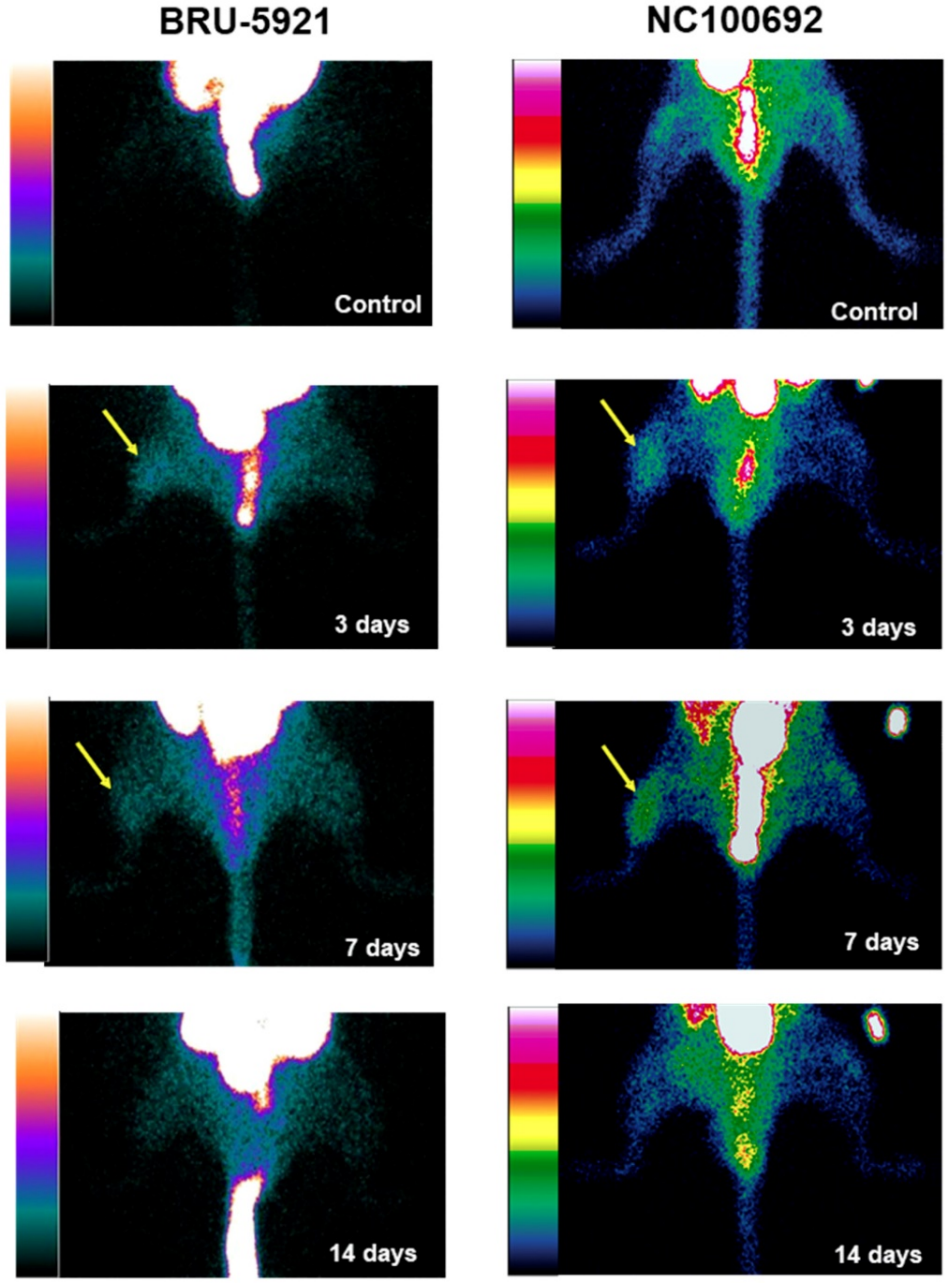
(Representative planar scintigraphic images of murine hindlimbs acquired at 75 min after injection of HIF-1α-targeted ^99m^Tc-BRU-5921 (left) or α_V_β_3_-targeted ^99m^Tc-NC100692 (right) prior to, as well as three, seven, and 14 days after surgical ligation. Arrows mark focal uptake of both radiotracers within the ischemic hindlimb.

The modalities used here enabled us to perform a series of quantitative imaging sessions designed to track the progress of individual animals as they recover from surgically-induced hindlimb ischemia. We successfully detected and quantified perfusion in both the deep and subcutaneous leg tissue and feet (see Figure 3 A, B, & C), as well as blood oxygenation in the deep leg tissue (Figure 3 D) over a range of timepoints spanning minutes to weeks after ligation. We also successfully quantified the activities of HIF-1α & *α_V_β_3_* at several key timepoints during recovery (see Figure 4 A & B). All told, these data reveal a series of interrelated biological events that punctuate the healing process—the landmarks of vascular recovery.

### Early Landmarks: Loss of Perfusion and Increased Hypoxia, and Transient Nitric Oxide Response

Naively, one might expect that perfusion should fall precipitously after surgery, and then stabilize until healing gets underway. While this is partly true—US and LSCI both show dramatic decreases in perfusion while PA shows a spike in hypoxia within the first 10 minutes (landmark 1; Figure 3 A-D)—we find that, with only a brief exception, perfusion continues to decrease gradually thereafter.

The exception occurs approximately 20 minutes post-surgery, at which time we observe a modest uptick in perfusion within the deep leg tissue (landmark 2; Figure 3 A). This uptick was apparent in almost every animal studied, although its duration varied between specimens (data not shown). One possible explanation may lie in a surge of endothelial nitric oxide (NO) production stimulated by the sudden onset of hypoxia. A complete understanding of the role of NO in vascular remodeling and angiogenesis remains elusive, and the available research can be somewhat contradictory 33-36. Nevertheless, previous studies have shown that: 1) NO modulates the consumption of oxygen when blood flow is restricted by competitive inhibition of cytochrome oxidase, and 2) hypoxic tissue increases expression of vascular endothelium growth factor (VEGF), which in turn gives rise to endogenous release of NO 37. Other pathways have also been implicated in the reactive hyperemia response, including those involving endothelium-derived relaxing factor (EDRF), a vasodilator with biological functions similar to NO 38-41, as well as certain pro-inflammatory pathways that have been shown to increase vascular permeability 42. Additional studies will be required to further establish the role of NO in the transient perfusion enhancement we observe, and to investigate possible therapeutic effects of NO on the ischemic microenvironment.

### Intermediate Landmarks: Early Stages of Vascular Recovery in the Subcutaneous Tissue

Following landmark 2, the US, LSCI and PA measurements show that perfusion and oxygenation continue to gradually fall in all tissues, taking at least an hour to reach a minimum in the shallow leg tissue (landmark 3, Figure 3 B), and even longer in the deep leg tissue and feet.

By the first day after surgery, however, we already observe signs of perfusion recovery in the shallow leg tissue (landmark 4, Figure 3 C). The simplest explanation for why the subcutaneous tissues begin to reperfuse earlier than the deeper tissues lies in their inherent physiological differences. Vascular density, metabolic behavior (including oxygen utilization), muscle fiber type and robustness to prolonged ischemia all vary considerably from one muscle group to the other within the hindlimb 43,44, and each of these can impact recovery. In addition to angiogenesis, there exists a secondary type of vascular remodeling which involves the growth of existing collateral arterioles into functional collateral arteries (Supplementary Results section *The dynamics of collateral blood vessels post-ischemia*, Figure S3). Dubbed arteriogenesis, this type of blood vessel growth is not regulated by hypoxia, but by the changes in sheer stress and inflammation that accompany the decrease in blood pressure associated with ligation 45-47. Because the vascular density in the surface tissue is generally quite high (presumably due to selection pressure favoring fast wound healing), arteriogenesis in these tissues can lead to the recovery of blood flow much faster than would otherwise be possible relying on angiogenesis alone.

### Late Landmarks: Molecular Signatures of Angiogenesis in the Deep Leg Tissue

The US and LSCI measurements show that perfusion in the deep tissue and feet continue to fall even while the shallow tissue begin to recover, ultimately reaching their lowest points approximately two days after surgery (landmark 5, Figure 3 A & C).

**Figure 5:**
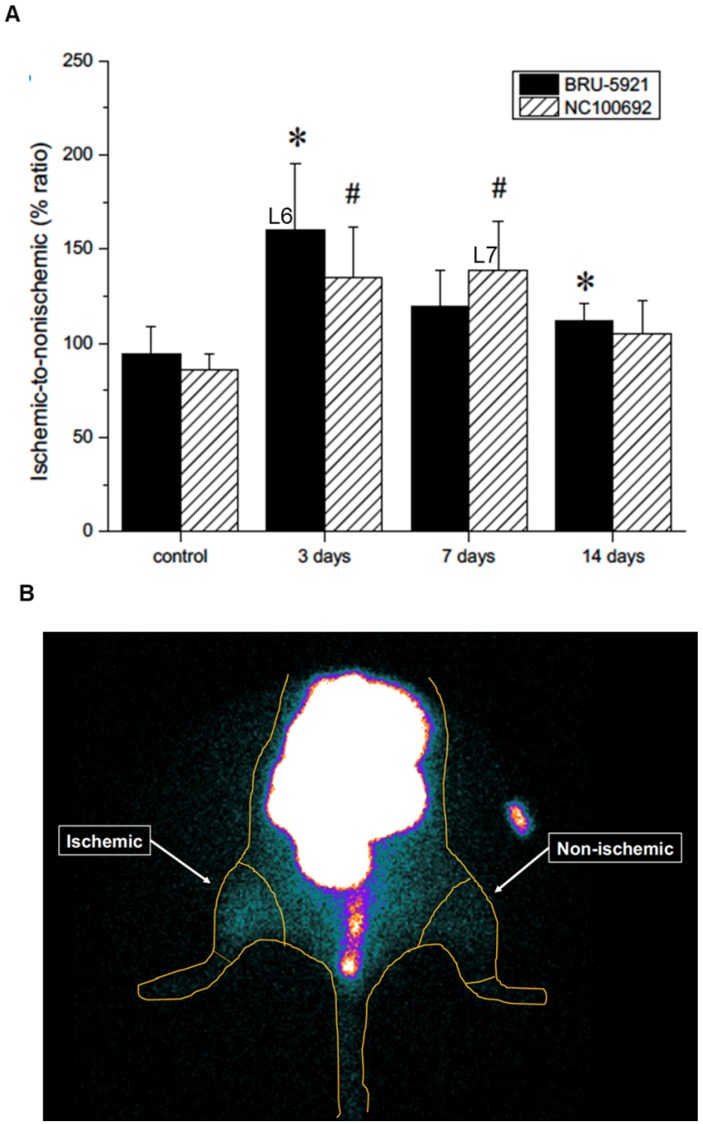
(A) Results from the image analysis using two-dimensional regions-of-interest (ROIs) drawn on planar scintigraphic images of ^99m^Tc-BRU-5921 (solid bars) and ^99m^Tc-NC100692 (dashed bars) prior to, as well as three, seven, and 14 days after ligation. Values were expressed as % ratio (ischemic to non-ischemic). Asterisks (*) indicate significant differences (p < 0.05) in measured ^99m^Tc-BRU-5921 activity values relative to the pre-ligation state, while number signs (**) indicate significant differences in ^99m^Tc-NC100692 activity. (B) Representative ROIs of scintigraphic images analysis. L above each time point indicates a landmark.

Shortly thereafter, angiogenesis begins in earnest. Around three days after surgery, we observe a peak in HIF-1α expression within the deep leg tissue (landmark 6, Figure 5 A)—a timing in agreement with other similar studies 48. The delayed onset may be due to the mechanism by which HIF-1α expression is regulated. Under normoxic conditions, oxygen-dependent post-translational modifications mark the protein for rapid degradation; during hypoxia, however, the protein cannot be modified and degraded 48,49, and as it gradually accumulates, it modulates the expression of dozens of genes, including *α_V_β_3_*. Indeed, we see enhanced *α_V_β_3_* activity after three days, and a peak in its activity around day seven (landmark 7, Figure 5 A).

We note also that both probes, ^99m^Tc-BRU-5921 and ^99m^Tc-NC100692, exhibited favorable organ excretion (Supplementary Results section *BRU-5921 and NC100692 exhibit favorable pharmacokinetics*, and Figure S4), and may find utility in future translational studies.

### Final Landmarks: Vascular Recovery in the Deep Leg Tissue and Feet

With angiogenesis in full swing a week after surgery, the deep tissues and feet begin to exhibit functional revascularization. On day seven, both US and LSCI show the first signs of reperfusion in these regions, and blood oxygenation appears to have returned to normalcy (landmark 8, Figure 3 A, C, & D). By the two-week timepoint, recovery is essentially complete; we observe a return to normal perfusion levels in the feet (landmark 9, Figure 3 C), and a leveling off of perfusion in the leg (Figure 3 A & B). We also observe considerable decreases in the expression levels of both HIF-1α and *α_V_β_3_* relative to their respective peaks days earlier (Figure 5 A), indicating that the hypoxic conditions that initially drove angiogenesis in the deep leg tissue have finally abated.

### *In vitro* postmortem evaluation of the ischemic microenvironment

**Figure 6:**
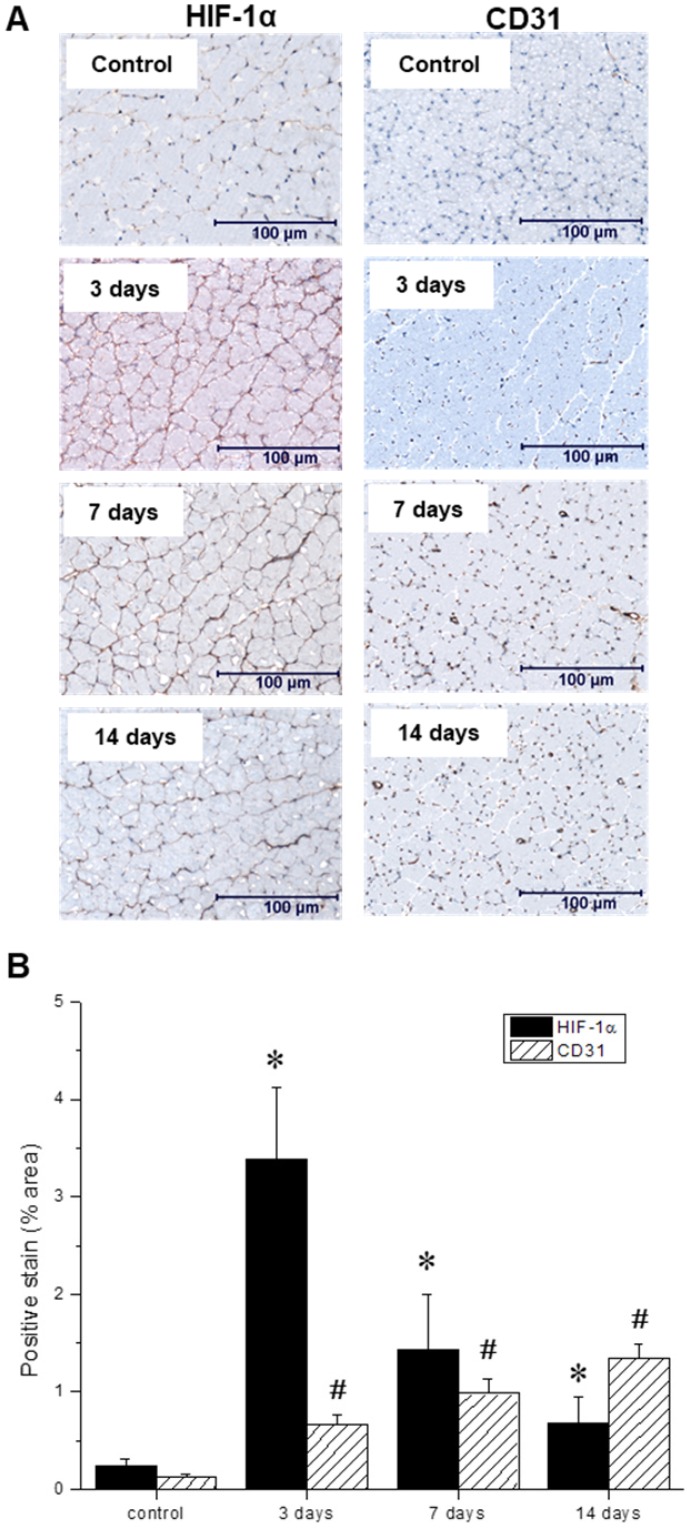
(A) Representative immunohistochemical microscopic images of gastrocnemius muscle collected from the ischemic hindlimb prior to (control), and at three, seven, and 14 days after ligation. Tissue samples were stained with primary antibodies against hypoxia-inducible factor-1 (HIF-1α, left) and platelet endothelial cell adhesion molecule, or PECAM (CD31, right). (B) Quantitative analysis of immunohistochemical microscopic images. Values were expressed as % area of positively stained tissue (HIF-1α shown in black, CD31 shown with diagonal lines). Asterisks (*) indicate significant differences (p < 0.05) in measured HIF-1α expression relative to the pre-ligation state, while number signs (#) indicate significant differences in CD31 activity.

**Figure 7:**
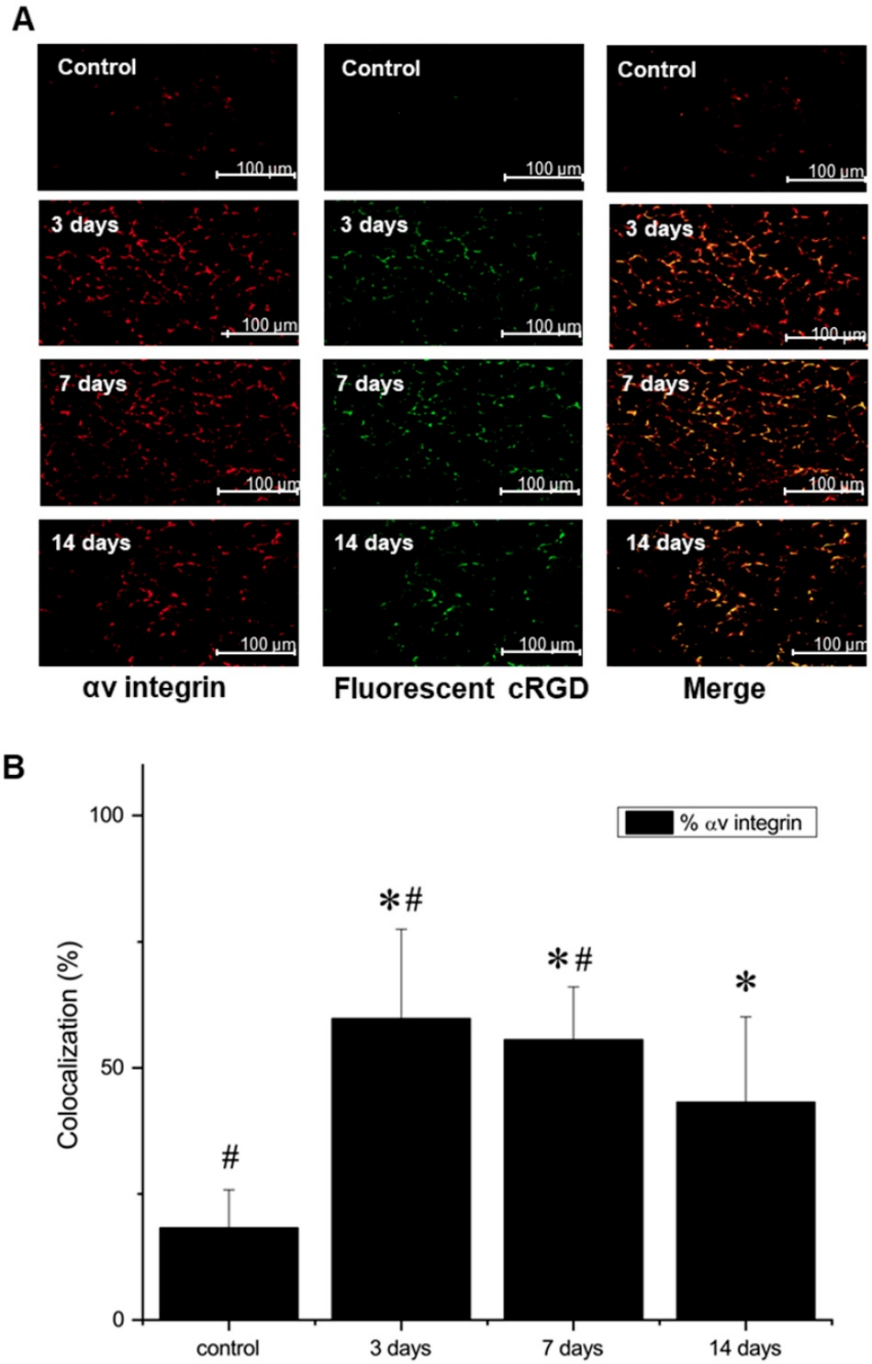
(A) Representative microscopic images of gastrocnemius muscle collected from ischemic hindlimb before (control), and at 3, 7, and 14 days after the surgical ligation of right femoral artery simultaneously incubated with an antibody against α_V_ integrin (left) and fluorescent FITC-labeled analogue of NC100692 (middle). Right column represents a result of merging of two-color channels (α_V_ integrin and fluorescent analogue of NC100692). (B) Quantitative analysis of co-localization between α_V_ integrin and fluorescent analogue of NC100692 in ischemic gastrocnemius muscle samples collected before (control) and at 3, 7, and 14 days after the surgical ligation of right femoral artery. Values are presented as percentage of cells expressing αV integrin co-localized with fluorescent FITC-labeled NC100692 analogue (solid bars). ∗P < 0.05 vs. control. #P < 0.05 vs. 14 days after ligation.

Throughout the multimodal serial imaging sessions, subsets of mice were sacrificed and their tissues were collected for subsequent immunohistochemistry and immunofluorescence analysis. Representative images are shown in Figures 6 A, and 7 A, respectively. Relative to the non-ischemic control samples, the ischemic tissues exhibited an approximately 14-fold surge in HIF-1α expression three days after ligation, which then gradually returned to baseline levels over the remainder of the experiment (Figure 6 B). Nevertheless, even at the 14-day timepoint, HIF-1α expression remained significantly above its basal level. In contrast, CD31 positive endothelial cells within the capillaries and small arterioles increased gradually over the course of the experiment, peaking at day 14.

Additional staining, using both AlexaFluor647-labeled anti-*α_V_* antibody and FITC-labeled NC100692, demonstrated not only a strong colocalization, indicating that NC100692 selectively targets cells engaged in angiogenesis, but also a rapid rise and subsequent slow decrease in the angiogenic response (see Figure 7 B).

## Discussion

Despite decades of research, the standard of care for patients with PAD has progressed little. This inability to translate discoveries from the bench to the clinic is due, at least in part, to inherent difficulties in assessing the efficacy of emerging treatments in a adequately holistic manner. Preclinical research often focuses on relatively few molecular or physiological markers, which may be measured at only one or a few time-points after treatment. Moreover, the lack of standardization in the type of measures and times chosen between studies renders cross-study comparisons of PAD treatments difficult or impossible. One study might report an enhancement in CD31^+^ cells following one type of treatment 50, while another might report increased markers of muscle respiration after some other type of treatment 51. Both measures may be perfectly reasonable given the focuses of their respective studies, but they are nevertheless hard to compare. Which therapy actually led to better outcomes for the mice? Which has the best chance of being effectively translated?

**Figure 8:**
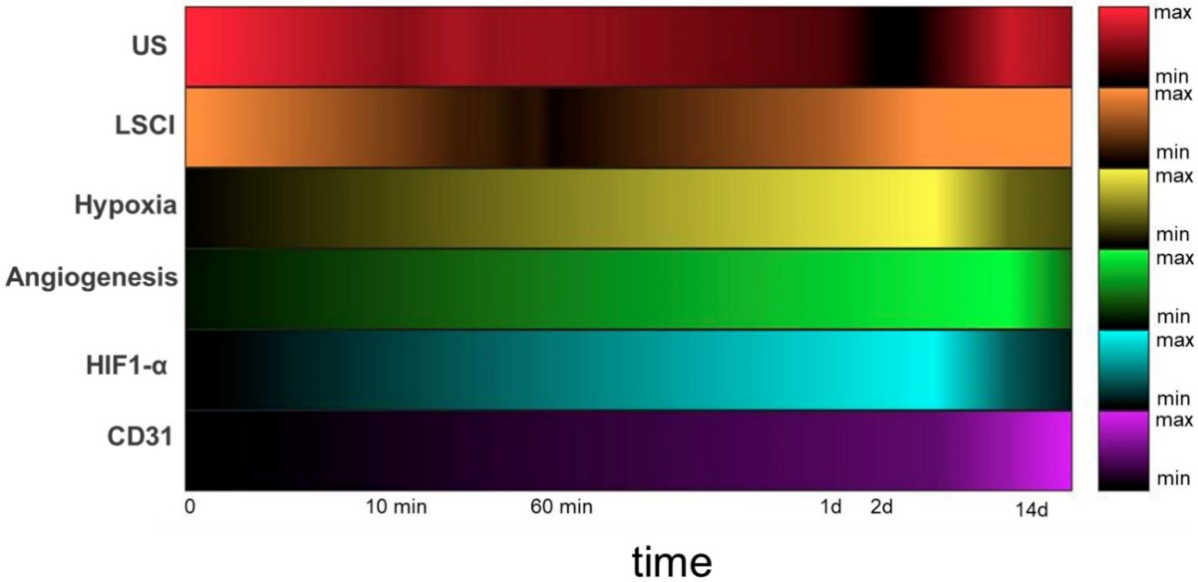
Heatmaps depicting measured circulatory parameters over a log-time axis. In each case, darker colors represent lower relative measurements, and the 0 timepoint indicates pre-ligation. While US (red) measures perfusion of the deep tissue (~ 6 mm), LSCI (orange) measures perfusion within 1mm of the tissue surface. Hypoxia (yellow) and α_V_β_3_ (green) expression were both measured using SPECT imaging with the targeted probes ^99m^Tc-BRU-5921 and ^99m^Tc-NC100692, respectively. HIF-1α (blue) and CD31 (purple) expression in ischemic tissue were quantified from immunohistochemistry images.

A shift toward multimodal imaging strategies has been underway for some time, driven largely by researchers' needs for tracking a number of different biological processes simultaneously 52,53. One of the goals of this work is to describe a new multidimensional platform capable of measuring several different molecular and physiological markers associated with vascular occlusion and subsequent neovascularization (see Table 1). The platform comprises a number of complementary imaging technologies, including scintigraphic imaging (in order to track angiogenesis), power Doppler US and LSCI (to detect changes in blood perfusion in the deep and shallow tissues of the leg and feet, respectively), and PA (to assess tissue oxygenation). Combined, these imaging methods allow us to follow the progress of each of the animals through their recovery from surgically induced hindlimb ischemia (Figure 8). Not only do they enable direct visualization of their respective targets, they also allow for their quantification. The US image analysis in particular employs state-of-the-art methods for detecting and quantifying slow perfusion—a capability critical for effective use in situations involving obstructed blood flow 16.

The multidimensional imaging platform paints a holistic and time-resolved portrait of vascular recovery, with several clearly-defined “landmarks” along the way. By adopting this series of landmarks as a standardized vocabulary for describing the impact of a given PAD treatment, the field can better evaluate each emerging treatment in the context of the others. A given therapy might, for example, shorten the time before HIF-1α activity peaks at landmark 6, or increase *α_V_β_3_* expression at landmark 7, but still not lead to earlier or more complete revascularization at landmark 9. Another therapy might lead to more modest changes in the earlier landmarks but may speed up the final ones. In either event, a well-defined map of these important biological events enables a better understanding of the strengths and weaknesses of each therapeutic approach and allows researchers to more effectively combine complementary treatments or improve upon existing ones in order to yield the next generation of PAD treatments. It's a powerful concept.

Finally, one of the strengths of the imaging platform is its extensibility for both research and clinical applications. Researchers interested in PAD-associated inflammation, endothelial proliferation, or VEGF signaling, as just a few examples, might include additional targeted molecular imaging sessions designed to track the expression levels of the receptor for advanced glycosylation end-products (RAGE) 54 CD105 55, CD146 56, or VEGFR 57, respectively. Similarly, the impact of preexisting conditions can be investigated using other mouse lines, such as db/db (for type II diabetes) or ApoE-/- (for atherosclerosis). The results of such studies, especially when presented within our existing framework of the landmarks of vascular recovery, would serve to further flesh out spatio-temporal interdependencies in the molecular events that drive cardiovascular disease and vascular recovery across a range of medical backgrounds. Not only can these leads to a deeper mechanistic understanding of the causes of PAD, it may also suggest new approaches to monitoring and treating the disease in humans.

## Conclusions

Fifteen years ago, a commentary by Belch *et al.* called for new diagnosis and treatment options for PAD patients 1. In the years since, hundreds of encouraging preclinical and clinical results have been reported, but few have been successfully translated, and none have become part of the standard of care for PAD patients. The field of therapeutic angiogenesis in particular, which a few decades ago showed so much promise, has yet to produce a single FDA-approved targeted treatment. Nevertheless, due to the prevalence and debilitating complications associated with PAD, the field remains active. What is desperately needed are new methods for quantifying the efficacies of the next generation of treatments in ways that facilitate cross-study comparisons. The work presented here is intended to do just that.

The multi-modal imaging strategy described here allows us not only to monitor the physiological response of mice to surgically-induced vascular occlusion, but also to quantify the timings of key events that transpire during the healing process. These “landmarks” can be used by researchers to help plan their experimental protocols, for example by establishing timeframes over which different measures of vascular function can be meaningfully assessed before they are obscured through the natural healing process. More importantly, these landmarks also provide a framework through which researchers can evaluate the onset, strength, and duration of a series of well-defined molecular and physiological events and be able to determine whether those changes actually lead to faster and/or more complete revascularization. In this way, multimodal imaging strategies like ours are poised to provide the PAD research community with a powerful new set of tools.

## Supplementary Material

Supplementary methods, results, figures and tables.

## Figures and Tables

**Table 1:** Selective characteristics for the components of the multimodal platform.

Imaging Modality	US (24MHz)	PA	LSCI	Scintigraphic Imaging
Depth	4-6 mm	3-4 mm	1 mm	No limit
Contrast Source	Endogenous	Endogenous	Endogenous	Exogenous
Pixel or Voxel representation	Echo power in dB scale	Optical absorption	Optical power in flux scale	Counts per second
Temporal res.	ms	s	s	min
Spatial res.	High	Moderate	Moderate	Low
Anatomy	√	√	√	-
Functional	√	√	√	√
Molecular	-	-	-	√

## References

[1] Belch JJ, Topol EJ, Agnelli G, Bertrand M, Califf RM, Clement DL, Creager MA, Easton JD, Gavin JR, Greenland P (2003). Critical Issues in Peripheral Arterial Disease Detection and Management: A Call to Action. Arch Gen Intern Med.

[2] Margolis J, Barron JJ, Grochulski WD (2005). Health Care Resources and Costs for Treating Peripheral Artery Disease in a Managed Care Population: Results from Analysis of Administrative Claims Data. J Manag Care Pharm.

[3] Swaminathan A, Vemulapalli S, Patel MR, Jones WS (2014). Lower Extremity Amputation in Peripheral Artery Disease: Improving Patient Outcomes. Vasc Health Risk Manag.

[4] Bashir R, Cooper CJ (2003). Evaluation and Medical Treatment of Peripheral Arterial Disease. Curr Opin Cardiol.

[5] Ekinci G, Bakir İ (2015). Evaluation of Publications on Cardiovascular and Peripheral Vascular Diseases Within the Frame of Scientific Publication Indicators. Interv Cardiol Rev.

[6] Folkman J (1995). Angiogenesis in Cancer, Vascular, Rheumatoid and Other Disease. Nat Med.

[7] Fam NP, Verma S, Kutryk M, Stewart DJ (2003). Clinician Guide to Angiogenesis. Circulation.

[8] Ahmed Z, Bicknell R (2009). Angiogenic Signalling Pathways. Angiogenesis Protocols.

[9] Cross MJ, Claesson-Welsh L (2001). FGF and VEGF Function in Angiogenesis: Signalling Pathways, Biological Responses and Therapeutic Inhibition. Trends Pharmacol Sci.

[10] Raghu Adya, Bee K Tan, Anu Punn (2007). Visfatin induces human endothelial vegf and mmp-2/9 production via mapk and pi3k/akt signalling pathways: novel insights into visfatin-induced angiogenesis. Cardiovasc Res.

[11] Krock BL, Skuli N, Simon MC (2011). Hypoxia-Induced Angiogenesis: Good and Evil. Genes & Cancer.

[12] Iyer SR, Annex BH (2017). Therapeutic Angiogenesis for Peripheral Artery Disease: Lessons Learned in Translational Science. JACC Basic Transl Sci.

[13] Demircioglu F, Hodivala-Dilke K (2016). αvβ3 Integrin and Tumour Blood Vessels—Learning from the Past to Shape the Future. Curr Opin Cell Biol.

[14] Raval Z, Losordo DW (2013). Cell Therapy of Peripheral Arterial Disease: From Experimental Findings to Clinical Trials. Circ Res.

[15] Waters RE, Terjung RL, Peters KG, Annex BH (2004). Preclinical Models of Human Peripheral Arterial Occlusive Disease: Implications for Investigation of Therapeutic Agents. J Appl Physiol.

[16] Kim M, Abbey CK, Hedhli J, Dobrucki LW, Insana MF (2017). Expanding Acquisition and Clutter Filter Dimensions for Improved Perfusion Sensitivity. IEEE Trans Ultrason Ferroelectr Freq Control.

[17] Cantisani V, Bertolotto M, Weskott H, Romanini L, Grazhdani H, Passamonti M, Drudi F, Malpassini F, Isidori A, Meloni F (2015). Growing Indications for Ceus: The Kidney, Testis, Lymph Nodes, Thyroid, Prostate, and Small Bowel. Eur J Radiol.

[18] Lin JB, Phillips EH, Riggins TE, Sangha GS, Chakraborty S, Lee JY, Lycke RJ, Hernandez CL, Soepriatna AH, Thorne BR (2015). Imaging of Small Animal Peripheral Artery Disease Models: Recent Advancements and Translational Potential. Int J Mol Sci.

[19] Nie L, Wang S, Wang X, Rong P, Ma Y, Liu G, Huang P, Lu G, Chen X (2014). *In vivo* Volumetric Photoacoustic Molecular Angiography and Therapeutic Monitoring with Targeted Plasmonic Nanostars. Small.

[20] Nie L, Huang P, Li W, Yan X, Jin A, Wang Z, Tang Y, Wang S, Zhang X, Niu G (2014). Early-Stage Imaging of Nanocarrier-Enhanced Chemotherapy Response in Living Subjects by Scalable Photoacoustic Microscopy. Acs Nano.

[21] Li W, Chen R, Lv J, Wang H, Liu Y, Peng Y, Qian Z, Fu G, Nie L (2018). *In vivo* Photoacoustic Imaging of Brain Injury and Rehabilitation by High-Efficient Near-Infrared Dye Labeled Mesenchymal Stem Cells with Enhanced Brain Barrier Permeability. Adv Sci.

[22] Jayanthy A, Sujatha N, Reddy MR, Narayanamoorthy V (2014). Non Invasive Blood Flow Assessment in Diabetic Foot Ulcer Using Laser Speckle Contrast Imaging Technique.

[23] Dobrucki LW, Muinck ED de, Lindner JR, Sinusas AJ (2010). Approaches to Multimodality Imaging of Angiogenesis. J Nucl Med.

[24] Dobrucki LW, Tsutsumi Y, Kalinowski L, Dean J, Gavin M, Sen S, Mendizabal M, Sinusas AJ, Aikawa R (2010). Analysis of Angiogenesis Induced by Local IGF-1 Expression After Myocardial Infarction Using microSPECT-Ct Imaging. J Mol Cell Cardiol.

[25] Haubner R, Kuhnast B, Mang C, Weber WA, Kessler H, Wester HJ, Schwaiger M (2004). [18F]Galacto-RGD: Synthesis, Radiolabeling, Metabolic Stability, and Radiation Dose Estimates. Bioconjug Chem.

[26] Iagaru A, Mosci C, Shen B, Chin FT, Mittra E, Telli ML, Gambhir SS (2014). (18)F-Fpprgd2 PET/CT: Pilot Phase Evaluation of Breast Cancer Patients. Radiology.

[27] Li ZB, Chen K, Chen X (2008). (68)Ga-Labeled Multimeric RGD Peptides for microPET Imaging of Integrin Expression. Eur J Nucl Med Mol Imaging.

[28] Meoli DF, Sadeghi MM, Krassilnikova S, Bourke BN, Giordano FJ, Dione DP, Su H, Edwards DS, Liu S, Harris TD, Madri JA, Zaret BL, Sinusas AJ (2004). Noninvasive Imaging of Myocardial Angiogenesis Following Experimental Myocardial Infarction. J Clin Invest.

[29] Hedhli J, Czerwinski A, Schuelke M, Płoska A, Sowinski P, La Hood L, Mamer SB, Cole JA, Czaplewska P, Banach M (2017). Synthesis, Chemical Characterization and Multiscale Biological Evaluation of a Dimeric-cRGD Peptide for Targeted Imaging of αvβ3 Integrin Activity. Sci Rep.

[30] Villanueva FS, Wagner WR (2008). Ultrasound Molecular Imaging of Cardiovascular Disease. Nat. Clin. Pract. Cardiovasc. Med.

[31] McDonald DM, Choyke PL (2003). Imaging of Angiogenesis: From Microscope to Clinic. Nat Med.

[32] Hua J, Dobrucki LW, Sadeghi MM, Zhang J, Bourke BN, Cavaliere P, Song J, Chow C, Jahanshad N, Royen N van, Buschmann I, Madri JA, Mendizabal M, Sinusas AJ (2005). Noninvasive Imaging of Angiogenesis with a 99m-Tc-Labeled Peptide Targeted at Integrin After Murine Hindlimb Ischemia. Circulation.

[33] Hopkins SP, Bulgrin JP, Sims RL, Bowman B, Donovan DL, Schmidt SP (1998). Controlled Delivery of Vascular Endothelial Growth Factor Promotes Neovascularization and Maintains Limb Function in a Rabbit Model of Ischemia. J Vasc Surg.

[34] Nanobashvili J, Neumayer C, Fuegl A, Punz A, Blumer R, Mittlböck M, Prager M, Polterauer P, Dobrucki LW, Huk I (2004). Combined L-Arginine and Antioxidative Vitamin Treatment Mollifies Ischemia-Reperfusion Injury of Skeletal Muscle. J Vasc Surg.

[35] Brevetti G, Schiano V, Chiariello M (2008). Endothelial Dysfunction: A Key to the Pathophysiology and Natural History of Peripheral Arterial Disease?. Atherosclerosis.

[36] Yu J, Zhuang Z, Drinane M, Kauser K, Rubanyi GM, Qian HS, Murata T, Escalante B, Sessa WC (2005). Endothelial Nitric Oxide Synthase Is Critical for Ischemic Remodeling, Mural Cell Recruitment, and Blood Flow Reserve. Proc Natl Acad Sci.

[37] Cleeter M, Cooper J, Darley-Usmar V, Moncada S, Schapira A and (1994). Reversible Inhibition of Cytochrome c Oxidase, the Terminal Enzyme of the Mitochondrial Respiratory Chain, by Nitric Oxide: Implications for Neurodegenerative Diseases. FEBS Let.

[38] Ho TK, Rajkumar V, Ponticos M, Leoni P, Black DCM, Abraham DJ, Baker DM (2006). Increased Endogenous Angiogenic Response and Hypoxia-Inducible Factor-1α in Human Critical Limb Ischemia. J Vasc Surg.

[39] Shen W, Xu X, Ochoa M, Zhao G, Bernstein R, Forfia P, Hintze T (2000). Endogenous Nitric Oxide in the Control of Skeletal Muscle Oxygen Extraction During Exercise. Acta Physiol.

[40] Huang AL, Silver AE, Shvenke E, Schopfer DW, Jahangir E, Titas MA, Shpilman A, Menzoian JO, Watkins MT, Raffetto JD (2007). Predictive Value of Reactive Hyperemia for Cardiovascular Events in Patients with Peripheral Arterial Disease Undergoing Vascular Surgery. Arterioscler Thromb Vasc Biol.

[41] Loscalzo J, Vita JA (1994). Ischemia, Hyperemia, Exercise, and Nitric Oxide. Circulation.

[42] You D, Waeckel L, Ebrahimian TG, Blanc-Brude O, Foubert P, Barateau V, Duriez M, LeRicousse-Roussanne S, Vilar J, Dejana E (2006). Increase in Vascular Permeability and Vasodilation Are Critical for Proangiogenic Effects of Stem Cell Therapy. Circulation.

[43] Mackie BG, Terjung RL (1983). Blood Flow to Different Skeletal Muscle Fiber Types During Contraction. Am J Physiol Heart Circ Physiol.

[44] Turóczi Z, Arányi P, Lukáts Á, Garbaisz D, Lotz G, Harsányi L, Szijártó A (2014). Muscle Fiber Viability, a Novel Method for the Fast Detection of Ischemic Muscle Injury in Rats. PloS One.

[45] Heil M, Schaper W (2007). Insights into Pathways of Arteriogenesis. Curr Pharm Biotechnol.

[46] Van Royen N, Piek JJ, Buschmann I, Hoefer I, Voskuil M, Schaper W (2001). Stimulation of Arteriogenesis; a New Concept for the Treatment of Arterial Occlusive Disease. Cardiovasc Res.

[47] Lee CW, Stabile E, Kinnaird T, Shou M, Devaney JM, Epstein SE, Burnett MS (2004). Temporal Patterns of Gene Expression After Acute Hindlimb Ischemia in Mice: Insights into the Genomic Program for Collateral Vessel Development. J Am Coll Cardiol.

[48] Walton HL, Corjay MH, Mohamed SN, Mousa SA, Santomenna LD, Reilly TM (2000). Hypoxia Induces Differential Expression of the Integrin Receptors αvβ3 and αvβ5 in Cultured Human Endothelial Cells. J Cell Biochem.

[49] Ke Q, Costa M (2006). Hypoxia-Inducible Factor-1 (Hif-1). Mol Pharmacol.

[50] Kalka C, Masuda H, Takahashi T, Kalka-Moll WM, Silver M, Kearney M, Li T, Isner JM, Asahara T (2000). Transplantation of Ex Vivo Expanded Endothelial Progenitor Cells for Therapeutic Neovascularization. J Cell Biochem.

[51] Baltgalvis KA, White K, Li W, Claypool MD, Lang W, Alcantara R, Singh BK, Friera AM, McLaughlin J, Hansen D (2014). Exercise Performance and Peripheral Vascular Insufficiency Improve with Ampk Activation in High-Fat Diet-Fed Mice. Am J Physiol Heart Circ Physiol.

[52] Xing Y, Zhao J, Conti PS, Chen K (2014). Radiolabeled Nanoparticles for Multimodality Tumor Imaging. Theranostics.

[53] Cai W, Chen X (2008). Multimodality Molecular Imaging of Tumor Angiogenesis. J Nucl Med.

[54] Konopka CJ, Wozniak M, Hedhli J, Ploska A, Schwartz-Duval A, Siekierzycka A, Pan D, Munirathinam G, Dobrucki IT, Kalinowski L (2018). Multimodal Imaging of the Receptor for Advanced Glycation End-Products with Molecularly Targeted Nanoparticles. Theranostics.

[55] Orbay H, Zhang Y, Hong H, Hacker TA, Valdovinos HF, Zagzebski JA, Theuer CP, Barnhart TE, Cai W (2013). Positron Emission Tomography Imaging of Angiogenesis in a Murine Hindlimb Ischemia Model with 64Cu-Labeled Trc105. Mol Pharm.

[56] Ferreira CA, Hernandez R, Yang Y, Valdovinos HF, Engle JW, Cai W (2018). ImmunoPET of Cd146 in a Murine Hindlimb Ischemia Model. Mol Pharm.

[57] Rodriguez-Porcel M, Cai W, Gheysens O, Willmann JK, Chen K, Wang H, Chen IY, He L, Wu JC, Li Z-b (2008). Imaging of Vegf Receptor in a Rat Myocardial Infarction Model Using PET. J Nucl Med.

