# Supplementary material for: Imaging the Landmarks of Vascular Recovery
Source: Theranostics. 2020 Jan 1;10(4):1733–45. doi: 10.7150/thno.36022 (PMC6993245; doi:10.7150/thno.36022)
Supplement: Supplementary file 1 — Supplementary methods, results, figures and tables. [file thnov10p1733s1.pdf]

Imaging the Landmarks of Vascular Recovery

Jamila Hedhli [a,e,1,*], MinWoo Kim [b,1], Hailey J. Knox [c,e], John A. Cole [d], Than Huynh [a], Matthew Schuelke [a,e], Iwona T. Dobrucki [e], Leszek Kalinowski [g,h], Jefferson Chan [c,e], Albert J. Sinusas [f], Michael F. Insana [a,e], Lawrence W. Dobrucki [a,e,h,*]

[a] Department of Bioengineering, University of Illinois at Urbana-Champaign, Urbana, IL

[b] Department of Electrical and Computer Engineering, University of Illinois at Urbana-Champaign, Urbana, IL

[c] Department of Chemistry, University of Illinois at Urbana-Champaign, Urbana, IL

[d] SimBioSys, Inc., Champaign, IL

[e] Beckman Institute for Advanced Science and Technology, Urbana, IL

[f] Department of Internal Medicine, Yale University School of Medicine, New Haven, CT

[g] Department of Medical Laboratory Diagnostics, Medical University of Gdansk, Poland

[h] Biobanking and Biomolecular Resources Research Infrastructure Poland (BBMRI.PL), Gdansk, Poland

*Corresponding authors:
Jamila Hedhli (hedhli2@illinois.edu) and Lawrence W. Dobrucki (dobrucki@illinois.edu)

[1]these authors contributed equally to this work

Correspondence and reprints: 405 N. Mathews Ave, MC-251, Urbana, IL 61801

Abstract

Background: Peripheral arterial disease (PAD) is a major worldwide health concern. Since the late 1990s therapeutic angiogenesis has been investigated as an alternative to traditional PAD treatments. Although positive preclinical results abound in the literature, the outcomes of human clinical trials have been discouraging. Among the challenges the field has faced has been a lack of standardization of the timings and measures used to validate new treatment approaches.

Methods: In order to study the spatiotemporal dynamics of both perfusion and neovascularization in mice subjected to surgically-induced hindlimb ischemia (n= 30), we employed three label-free imaging modalities (a novel high-sensitivity ultrasonic Power Doppler methodology, laser speckle contrast, and photoacoustic imaging), as well as a tandem of radio-labeled molecular probes, ^{99m}Tc-NC100692 and ^{99m}Tc-BRU-5921 respectively, designed to detect two key modulators of angiogenic activity, $\alpha_V\beta_3$and HIF-1α , via scintigraphic imaging.

Results: The multimodal imaging strategy reveals a set of “landmarks”—key physiological and molecular events in the healing process—that can serve as a standardized framework for describing the impact of emerging PAD treatments. These landmarks span the entire process of neovascularization, beginning with the rapid decreases in perfusion and oxygenation associated with ligation surgery, extending through pro-angiogenic changes in gene expression driven by the master regulator HIF-1α , and ultimately leading to complete functional revascularization of the affected tissues.

Conclusions: This study represents an important step in the development of multimodal non-invasive imaging strategies for vascular research; the combined results offer more insight than can be gleaned through any of the individual imaging methods alone. Researchers adopting similar imaging strategies and will be better able to describe changes in the onset, duration, and strength of each of the landmarks of vascular recovery, yielding greater biological insight, and enabling more comprehensive cross-study comparisons. Perhaps most important, this study paves the road for more efficient translation of PAD research; emerging experimental treatments can be more effectively assessed and refined at the preclinical stage, ultimately leading to better next-generation therapies.

Keywords: Angiogenesis, hypoxia, hindlimb ischemia , ^{99m}Tc-NC100692, ^{99m}Tc-BRU-5921, perfusion, functional recovery, Power Doppler imaging.

Supplementary Information Methods

Animals

30 male black C57BL/6 mice (Charles River Laboratories, US) were anesthetized with 1-3% isofluorane. The right femoral artery was exposed and two ligatures were placed distally to the profundus branch and proximally from the artery bifurcation. The vessel between the ligatures was then cut using a coagulation pen. On the opposite leg, a sham operation was performed which included a skin incision, and exposure of the left femoral artery, but no ligatures were placed and the vessel was not cut. The surgical model used in all experiments resulted in small postoperative wounds with minimal inflammation, and the formation of collaterals between several proximal vessels including the profundus branch and the distal section of the femoral artery.

Ultrasound Imaging

Echo data were acquired using a Vevo 2100 system and an MS 400 linear-array transducer (FUJIFILM VisualSonics Inc. Toronto, Ontario, Canada). The transducer, emitting 2-cycle pulses with a 24 MHz center frequency, was positioned on the ischemic region of each animal's hindlimb. The focal depth was set between 4 & 6 mm, and the aperture size was adjusted to maintain f/2. Color-Doppler acquisition frames were comprised of a packet of 17 pulses transmitted at a time interval of 1 ms, giving a slow-time pulse repetition frequency (PRF) of 1 kHz. For each pulse transmission, beam formed IQ echo data were recorded for off-line analysis. The scan-lines were separated laterally by 0.06 mm. For each animal, 100 Doppler frames were recorded with a frame-time interval of 0.11s. The complex-valued IQ echo data are represented by a 3-D data array, $\boldsymbol{\mathcal{X}} \in \mathbb{C}^{N\times S\times K}$, where N, S and K are the numbers of slow-time samples, spatial samples, and frames, respectively (see Figure 1).

A filtering procedure was applied to the echo data to form Power Doppler images and to isolate blood components from unwanted clutter (*e.g.* surrounding tissue motion and acquisition noise). We adopt a block-wise method to enhance the filter performance. This technique is robust to the high degree of complexity associated with the spatiotemporally-varying characteristics of the data sources. The full data array $\boldsymbol{\mathcal{X}}$ are divided into overlapping sub-arrays $\widetilde{\boldsymbol{\mathcal{X}}}_j \in \mathbb{C}^{\dot{N}\times\dot{S}\times\dot{K}}$, $j \in \{1,\cdots,J\}$, where J is the total number of blocks. Each sub-array is individually processed by a higher-order singular value decomposition (HOSVD) technique to detect the local perfusion. The HOSVD of $\widetilde{\boldsymbol{\mathcal{X}}}_j$ is expressed as

$$\widetilde{\boldsymbol{\mathcal{X}}}_j = \sum_{(i_1,i_2,i_3)\in\Omega} g_{i_1,i_2,i_3}\,\mathbf{u}_{i_1}\times\mathbf{v}_{i_2}\times\mathbf{w}_{i_3},$$

where Ω signifies full space and $\times$ is an outer-product operation. The vectors $\mathbf{u}_{i_1}$, $\mathbf{v}_{i_2}$ and $\mathbf{w}_{i_3}$ are i_1, i_2 and i_3th eigenvectors for the slow-time, spatial and frame-time modes, respectively. The new coordinates, by means of combination of the eigenvectors, naturally give rise to a separation of the sources through their statistical independence. g_{i_1,i_2,i_3} is associated with signal power at (i_1, i_2, i_3)th coordinates. The filtering is

performed by selecting the subspace where blood dominates and rejecting the rest where clutter and noise dominates. The process can be expressed as

$$\widetilde{\boldsymbol{\mathcal{B}}_j} = \sum_{(i_1,i_2,i_3)\in B} g_{i_1,i_2,i_3}\,\mathbf{u}_{i_1} \times \mathbf{v}_{i_2} \times \mathbf{w}_{i_3},$$

where $\widetilde{\boldsymbol{\mathcal{B}}_j}$ is the filtered data and B is the blood subspace. The subspace is roughly determined by prior knowledge of properties of the sources. The optimal one is empirically selected by comparison of the resulting image contrast. The local spatial perfusion is estimated by averaging signal power $|\widetilde{\boldsymbol{\mathcal{B}}_j}|^2$ over slow-time and frame-time axes, and converting the result to dB-scale. The final image is obtained by compounding all J local estimates (Figure 1). See Table 1 summarizing all parameter values.

Laser Speckle Contrast Imaging

LSCI images were obtained using a moorFLPI-2 laser perfusion imager (Moor Instrument, UK) operated from a Windows-based computer system installed with the moorFLPI software (moorFLPI Measurement V3.0, Moor Instruments, Devon, UK). Before initiating measurements, the laser speckle instrument was calibrated according to the manufacturer’s recommendations. Then, a divergent beam (785 nm, near-infrared) illuminated hindlimb tissue to a 1 mm in depth. High-resolution speckle images were acquired using a 768 × 576 pixel grayscale charge-coupled device camera set to record each frame for a duration of 60 seconds. In addition, the feet of three (n=3) mice were imaged prior to surgery and at 1 hr, 1 day, 2 days and 7 days post-surgery. For collateral circulation assessment, the skin was removed from the right hindlimb of the animal. The mice were imaged prior to surgery, and subsequently at 2,10, and 25 min post-ligation.

Photoacoustic Imaging

Photoacoustic imaging was performed with an Endra Nexus 128 PA tomographer (Endra, Inc., Ann Arbor, MI, USA) at 20, 30, 40, 50, and 60 minutes, and 1, 2, and 7 days post-surgery. Images were acquired at 750 and 850 nm using continuous rotation mode with a 6 second rotation time. A 3D reconstruction was performed for each image, and signal intensity was accumulated over an 8 mm thick slab. The signal in each PA image was quantified by determining the mean PA signal in a 2 mm^2 region of interest (ROI). A ratiometric signal was determined by taking the ratio of signal at 750 nm to 850 nm. Data was processed using the freely available Horos software (Horos Project, https://www.horosproject.org/).

Perfusion Trend Analysis for Ultrasound and Laser Speckle Contrast Imaging

Perfusion images obtained through Power Doppler US were used to track tissue perfusion changes over the course of two weeks. Through imaging the animal hindlimbs at various time points (before and after ligation), we were able to track the average perfusion changes over the regions illustrated in Figure [USDanalysis]. These regions

intentionally exclude arteries in order to focus on tissue perfusion. The depth of each region was set to less than 6 mm to provide a consistently high echo signal to noise ratio. The average perfusion during the t-th time point in the m-th mouse is represented as

$$p_{t,m}, t \in \{1, \cdots, T\}, m \in \{1, \cdots, M\},$$

where T and M are the number of time points and mice included in this study, respectively. We compute the change in perfusion over time as $q_{t,m} = p_{t,m} - p_{1,m}$ where $p_{1,m}$ is the preligation regional perfusion value. The temporal variation is analyzed by plotting sample mean $\overline{q}_t = \frac{1}{M}\sum_{m=1}^{M} q_{t,m}$ and standard error of the mean (SEM) $\tilde{q}_t = \sqrt{\frac{1}{M}}\sqrt{\frac{1}{M-1}\sum_{m=1}^{M}(q_{t,m} - \overline{q}_t)^2}$ for every time point t.

The trend for LSCI was obtained by a comparable procedure, but we report the ratio of signal from the right ischemic hindlimb to that of the left control hindlimb:

$$r_{t,m} = \langle p_{t,m}^{(R)} \rangle / \langle p_{t,m}^{(L)} \rangle$$

where $\langle p_{t,m}^{(R)} \rangle$ and $\langle p_{t,m}^{(L)} \rangle$ are perfusion averages over the boxes on right and left hindlimbs, respectively. Because the spatial absolute image values are highly sensitive to ambient light conditions we normalize the perfusion change using $g_{t,m} = r_{t,m} - r_{1,m} + 1$ and plot the mean and SEM for $g_{t,m}$ at every time point.

Blood Oxygenation Analysis for Photoacoustic Imaging

Because the oxygenated $(\mathrm{HbO_2})$ and deoxygenated (HbR) forms of hemoglobin have different near-infrared absorption properties, PA is able to detect the relative level of blood oxygenation [1]. Briefly, the amplitude of the acquired PA image describes the local energy absorption, $\phi_{t,m}(\lambda_i, x, y, z)$, at each time t and for each mouse m, measured at each wavelength λ_i (where $i \in \{1, \cdots, L\}$). Taking a spatial average then yields $\overline{\phi}_{t,m}(\lambda_i)$. The contribution of the two forms of hemoglobin to the spatial average absorption can be represented as

$$\overline{\phi}(\lambda_i) = \epsilon_{HbR}(\lambda_i)[\mathrm{HbR}] + \epsilon_{HbO_2}(\lambda_i)[\mathrm{HbO_2}]$$

where ϵ_{HbR} and ϵ_{HbO_2} are the molar extinction coefficients of HbR and $\mathrm{HbO_2}$ at wavelength λ_i, respectively, and square brackets denote the concentrations of oxygenated and deoxygenated hemoglobin. The two concentrations can thus be estimated by a least squares solution as

$$\begin{bmatrix} [\mathrm{HbR}]_{t,m} \\ [HbO2]_{t,m} \end{bmatrix} = \begin{bmatrix} \epsilon_{HbR}(\lambda_1) & \epsilon_{HbO_2}(\lambda_1) \\ \vdots & \vdots \\ \epsilon_{HbR}(\lambda_L) & \epsilon_{HbO_2}(\lambda_L) \end{bmatrix}^{\dagger} \begin{bmatrix} \overline{\phi}_{t,m}(\lambda_1) \\ \vdots \\ \overline{\phi}_{t,m}(\lambda_L) \end{bmatrix}$$

where $A^{\dagger} = (A^T A)^{-1} A^T$ denotes pseudo-inverse of matrix A. The relative change in oxygenation is consequently given as $\eta_{t,m} = [\mathrm{HbR}]_{t,m}/[\mathrm{HbO_2}]_{t,m}$. Three wavelengths, λ_1=750 nm, λ_2=800 nm, and λ_3=850 nm were used to reduce the estimation error, although two measurements could have sufficed in order to determine the concentrations. We used molar extinction coefficients from [2]. A total of seven (7) mice were scanned at several time points before and after ligation. We computed each ratio $\eta_{t,m}$, and finally plotted the sample mean and SEM for every time point to trace the change.

Serial Scintographic Imaging

All animals were laid in a supine position with legs fully extended and immobilized on a polyacrylic board. Animals were then injected intravenously with 1.5 ± 0.2 mCi of the targeting agent on 0, 3, 7 and 14 days of hindlimb ischemia and subjected to dynamic pinhole planar imaging. Planar imaging utilizes a gamma camera (VariCam, GE Healthcare) set with a large field of view and a pinhole collimator coupled to a 1-mm aperture and 15% energy window centered at 140 keV. At 75 minutes post-injection of the radiotracer, a static 15-minute image was acquired.

Histological Validation

Immediately after the completion of each scintographic imaging session, a subset of animals (n=3) were euthanized and tissue samples were collected for gamma well counting for biodistribution and processed for immunostaining. Their gastrocnemius/soleus muscle complexes were excised, embedded in TissueTec (Sakura, USA) and snap frozen in -150^{0}C methylbutane. Frozen sections (5 nm) were placed on microscope slides, fixed with pre-cooled acetone, and stored at -80^{0}C before staining. To evaluate skeletal muscle vascularity, muscle samples were stained with either an endothelial cell-specific marker anti-mouse CD31 Antibody (BD Pharmingen) or hypoxia specific marker anti-HIF-1α (BD Pharmingen). To assess probe specificity (FITC-labeled NC100692), muscle samples were stained with both AlexaFluor647-labeled anti-α_V (Abcam, USA) antibody and FITC-labeled NC100692. All staining procedures were performed according to the product-specific protocols. The stains for HIF-1α and CD31 were quantified for extent (percentage area) of positive staining in randomly chosen high-powered (200 $\times$) fields using algorithms validated by our group previously [3]. Colocalization analysis of the histological images was performed using the Fiji image processing package. Correlated α_V-stained and FITC-labeled NC100692-stained images were analyzed using the Coloc 2 plugin of ImageJ software package. Using this plugin, Manders' coefficients were calculated using Costes method for thresholding [4]. Three images per animal were analyzed.

Biodistribution

For biodistribution studies, a subset of animals (n=6) for ^{99m}Tc-BRU-5921 and (n=5) for ^{99m}Tc-NC100692 were euthanized and selected organ samples were collected for gamma well counting analysis. The weight and the ^{99m}Tc radioactivity of each tissue

sample was measured using a gamma well counter (Cobra Packard). The activity measured was corrected for background, decay time, and sample weight.

Supplementary Information Results

The dynamics of collateral blood vessels post-ischemia

To illustrate the transient behavior of blood perfusion found with LSCI, we imaged the collateral blood vessels near the profundus branch before and after ligation. Following the surgery, we observed an abrupt increase in the perfusion of many collateral vessels near the ligation site (see Figure 3). This indicates that these smaller vessels, which under normal circumstances play a relatively minor role in blood perfusion, can rapidly increase their flow rate in order bypass a vascular occlusion and maintain downstream blood delivery.

BRU-5921 and NC100692 exhibit favorable pharmacokinetics

To demonstrate that the radiotracers exhibit favorable organ excretion for the purposes of imaging hypoxia-induced peripheral neovascularization, we performed biodistribution studies 75 minutes after injection of ^{99m}Tc-NC100692 (for angiogenesis) and ^{99m}Tc-BRU-5921 (for hypoxia). ^{99m}Tc-BRU-5921 showed high activity in the kidneys, and substantial accumulation in both the liver and pancreas, whereas ^{99m}Tc-NC100692 demonstrated highest retention in the kidneys and little activity in other organs (Figure 4).

Table

Parameters

Parameter	Value
Axial (fast-time) samples (M)	200-272
Axial sampling rate	24.0 MHz
Axial size	6.4-8.7 mm
Slow-time samples (N)	17
Slow-time sampling rate	1.0 kHz
Frame-time samples (K)	100
Frame-time sampling rate	9 Hz
Scan-line numbers (L)	240-250
Scan-line density	16.67 lines/mm
Lateral size	14.4-15.0 mm
Spatial samples (S=LM)	48000-68000
Sub-block size ($\dot{N} \times \dot{S} \times \dot{K}$)	17×768×30
Slow-time division (J_1)	1
Spatial division (J_2)	1600
Frame-time division (J_3)	10
Sub-block numbers (J=$J_1J_2J_3$)	16000

Figure Legends

Figure 1: Ultrasound imaging and data analysis protocol. An anesthetized mouse is placed in a supine position and its right femoral artery is ligated to induce ischemia. Without contrast-enhancement, standard Doppler ultrasound acquisition results at 24MHz in 3D data array involving spatial, slow-time (kHz-order sampling rate), and frame-time (Hz-order sampling rate) axes. The data array is divided into sub-blocks and each data block is individually processed by the HOSVD methodology [5] to detect local blood perfusion.

Figure 2: Perfusion quantification using US and LSCI. US and LSCI images were obtained at multiple time points before and after femoral artery ligation. The white rectangular windows in the images represent individual ROIs over which perfusion was averaged. In the US images, the difference between the pre- and post-operative state was computed for each mouse sample. Subsequently, the mean and SEM of this difference was plotted at each time point. The graph shows 0 dB pre-ligation ($i = 1$) as a reference, and the relative changes for all post-ligation timepoints ($i > 1$). In the LSCI images, the ratio between an ischemic right hindlimb region and its corresponding healthy left hindlimb region was computed, and their difference (plus unity, such that all values are relative to 1) was plotted.

Figure 3: Representative LSCI collateral imaging data presented at a series of time points (prior to and following ligation). (A) An intact femoral artery and profundus branch prior to ligation. (B)-(D) The same regions two, 10, and 25 minutes after ligation. An immediate halt in blood flow via the femoral artery can be seen after two minutes. Within the first 10 minutes, a transient increase in collateral blood perfusion can be observed followed by decrease at minute 25. In the images, ‘pF’ indicates proximal femoral artery, ‘P’ indicates the profundus branch of femoral artery, and ‘dF’ indicates the distal femoral artery; white arrows indicate collateral blood vessels that help to maintain perfusion after ligation.

Figure 4: Biodistributions of ^{99m}Tc-BRU-5921 and ^{99m}Tc-NC100692 in selected organs at 75 min after intravenous injection. The principal clearance route for ^{99m}Tc-NC100692 was through urination, as evidenced by a very high radiotracer uptake in both kidneys. ^{99m}Tc-BRU-5921 was also principally excreted via urination, although it took somewhat longer, as evidenced by its greater liver retention.

Figures

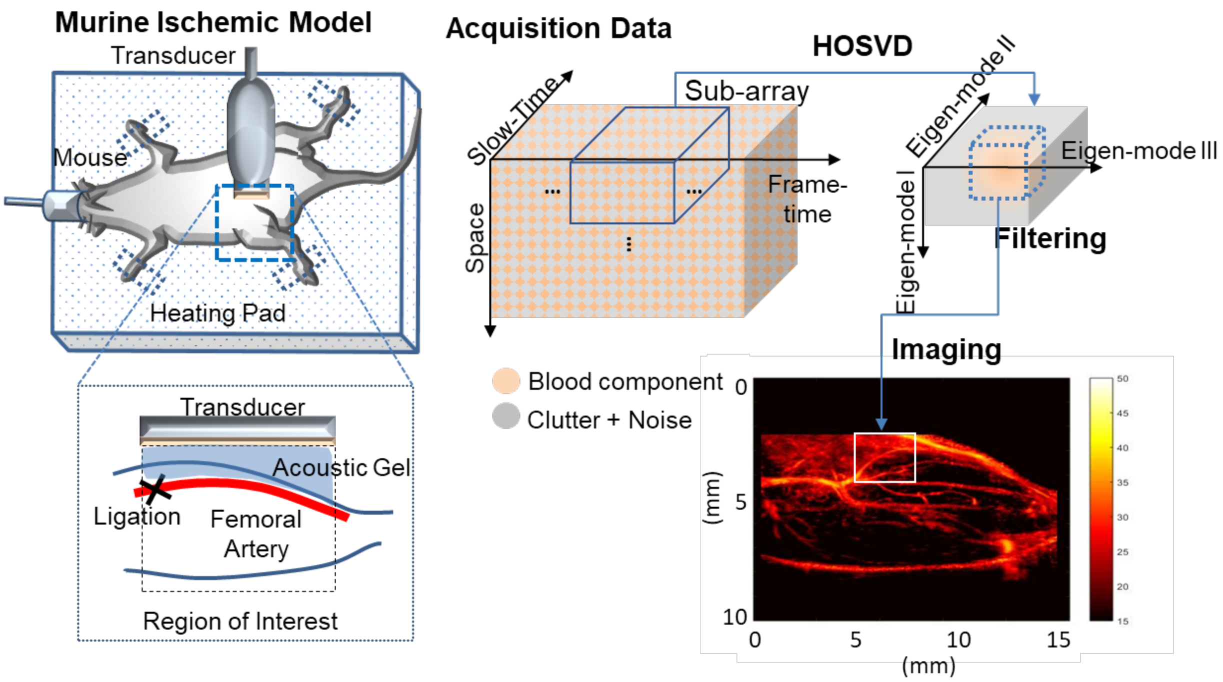

Figure 1

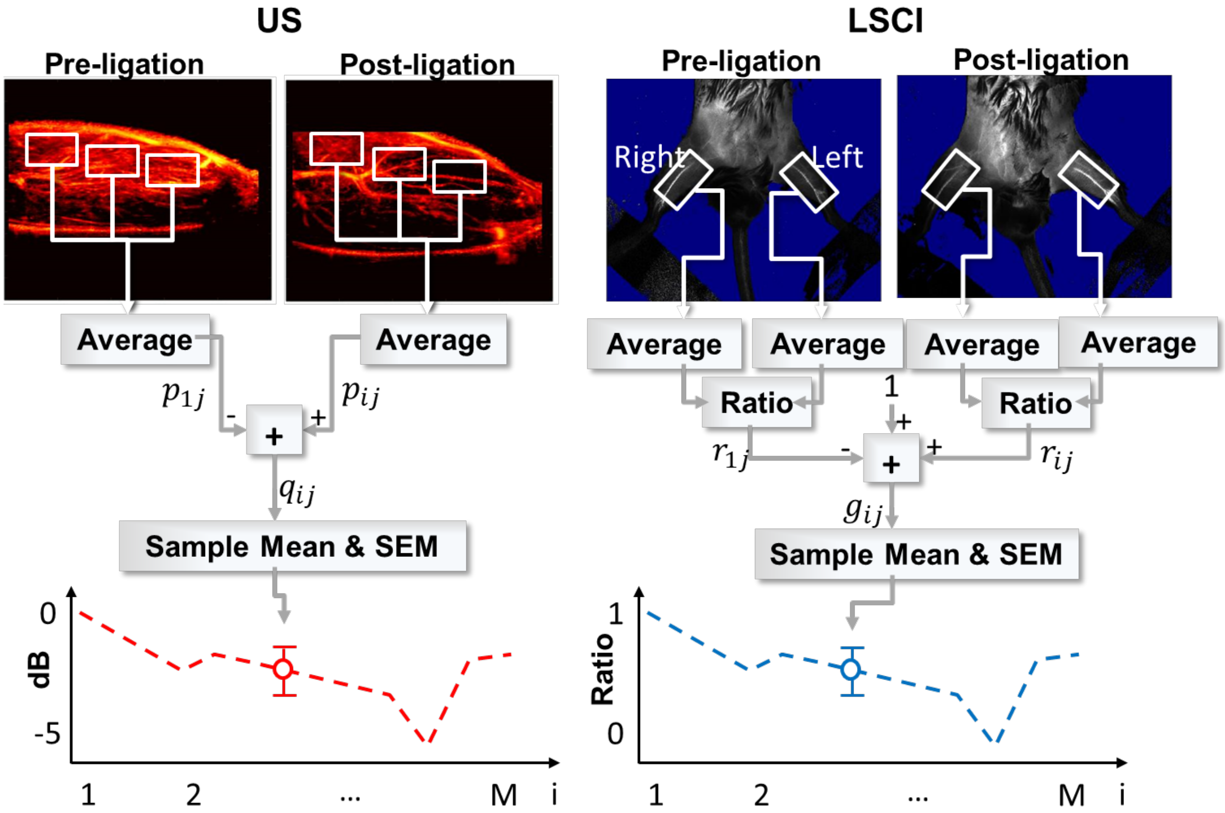

US
Pre-ligation
Post-ligation
Average
Average
p_{1j}
p_{ij}
-
+
+
q_{ij}
Sample Mean & SEM
0
dB
-5
1
2
...
M
i
LSCI
Pre-ligation
Post-ligation
Right
Left
Average
Average
Average
Average
Ratio
Ratio
1
+
r_{1j}
-
+
+
r_{ij}
g_{ij}
Sample Mean & SEM
1
Ratio
0
1
2
...
M
i

Figure 2

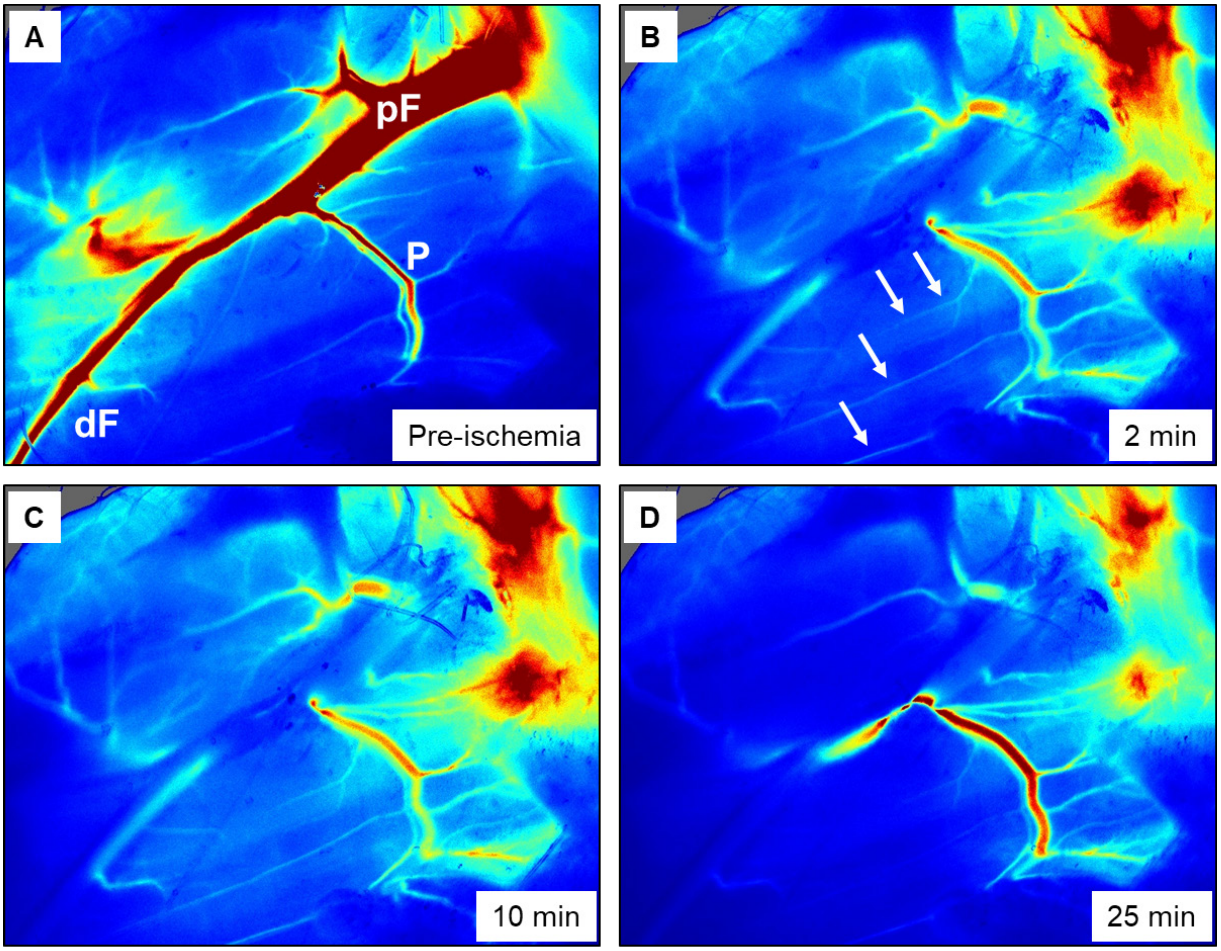

Figure 3

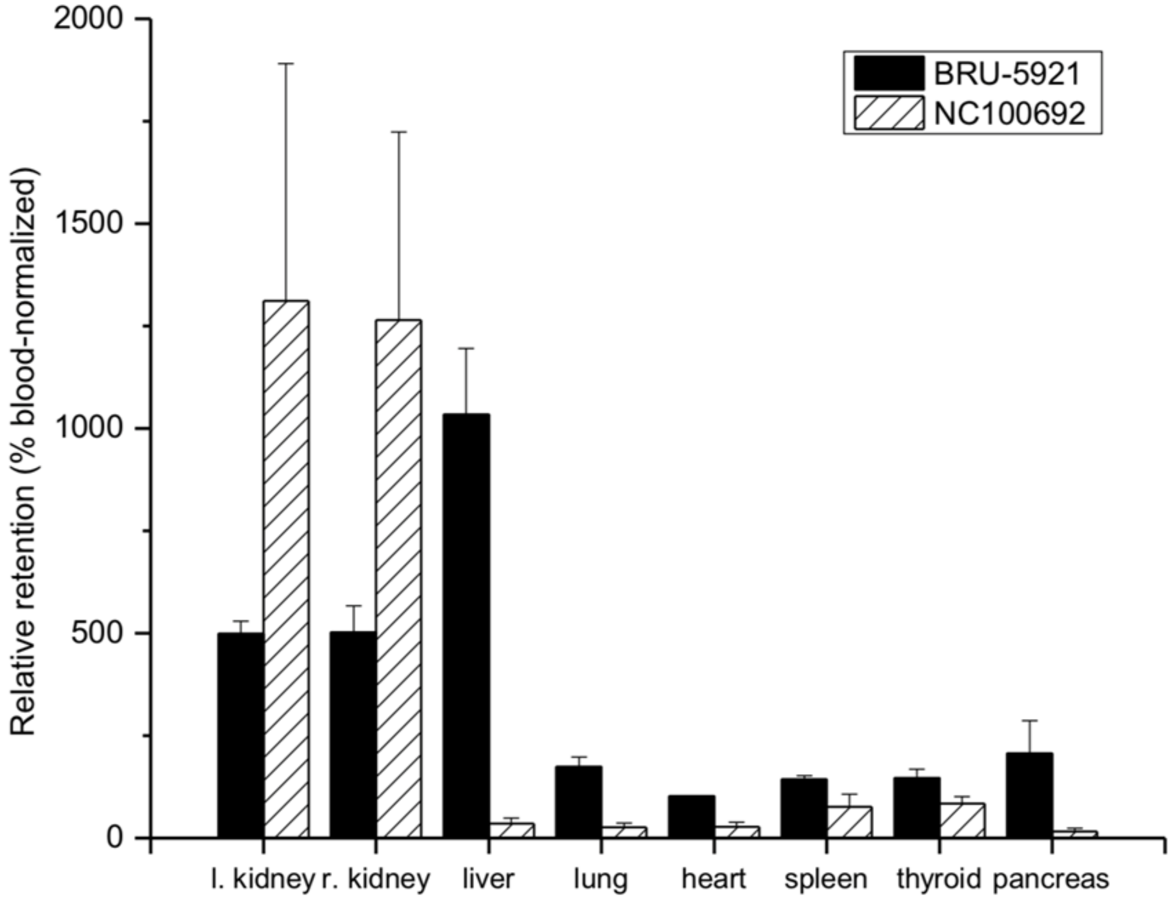

2000
1500
1000
500
0
Relative retention (% blood-normalized)
BRU-5921
NC100692
l. kidney
r. kidney
liver
lung
heart
spleen
thyroid
pancreas

Figure 4

Bibliography

[1] Cao F, Qiu Z, Li H, Lai P. Photoacoustic Imaging in Oxygen Detection. Appl Sci. 2017; 7(12): 1262.

[2] Prahl S. Tabulated Molar Extinction Coefficient for Hemoglobin in Water. 1999; URL https://dx.doi.org/10.1016/0014-5793(94)00424-2.

[3] Hua J, Dobrucki LW, Sadeghi MM, Zhang J, Bourke BN, Cavaliere P, Song J, Chow C, Jahanshad N, Royen N van, Buschmann I, Madri JA, Mendizabal M, Sinusas AJ. Noninvasive Imaging of Angiogenesis with a ^{99m}-Tc-Labeled Peptide Targeted at Integrin After Murine Hindlimb Ischemia. Circulation. 2005; 111(24): 3255–60.

[4] Manders E, Verbeek F, Aten J. Measurement of Co-Localization of Objects in Dual-Colour Confocal Images. J Microsc. 1993; 169(3): 375–382.

[5] Kim M, Abbey CK, Hedhli J, Dobrucki LW, Insana MF. Expanding Acquisition and Clutter Filter Dimensions for Improved Perfusion Sensitivity. IEEE Trans Ultrason Ferroelectr Freq Control. 2017; 64(10): 1429–1438.
